# Revealing global risks of labor abuse and illegal, unreported, and unregulated fishing

**DOI:** 10.1038/s41467-022-28916-2

**Published:** 2022-04-05

**Authors:** Elizabeth R. Selig, Shinnosuke Nakayama, Colette C. C. Wabnitz, Henrik Österblom, Jessica Spijkers, Nathan A. Miller, Jan Bebbington, Jessica L. Decker Sparks

**Affiliations:** 1 Stanford Center for Ocean Solutions, Stanford University, Stanford, USA; 2 Institute for the Oceans and Fisheries, University of British Columbia, Vancouver, Canada; 3 Stockholm Resilience Centre, Stockholm University, Stockholm, Sweden; 4 Graduate School of Agricultural and Life Sciences, The University of Tokyo, Tokyo, Japan; 5 ARC Centre of Excellence for Coral Reef Studies, James Cook University, Townsville, Australia; 6 Global Fishing Watch, Washington, DC USA; 7 The Pentland Centre for Sustainability in Business, Lancaster University, Lancaster, UK; 8 University of Nottingham Rights Lab, Highfield House, Nottingham, UK

**Keywords:** Sustainability, Ocean sciences, Geography

## Abstract

Labor abuse on fishing vessels and illegal, unreported and unregulated (IUU) fishing violate human rights, jeopardize food security, and deprive governments of revenues. We applied a multi-method approach, combining new empirical data with satellite information on fishing activities and vessel characteristics to map risks of labor abuse and IUU fishing, understand their relationships, and identify major drivers. Port risks were globally pervasive and often coupled, with 57% of assessed ports associated with labor abuse or IUU fishing. For trips ending in assessed ports, 82% were linked to labor abuse or IUU fishing risks. At-sea risk areas were primarily driven by fishing vessel flags linked to poor control of corruption by the flag state, high ownership by countries other than the flag state, and Chinese-flagged vessels. Transshipment risk areas were related to the gear type of fishing vessels engaged in potential transshipment and carrier vessel flags. Measures at port offer promise for mitigating risks, through the Port State Measures Agreement for IUU fishing, and ensuring sufficient vessel time at port to detect and respond to labor abuse. Our results highlight the need for coordinated action across actors to avoid risk displacement and make progress towards eliminating these socially, environmentally and economically unsustainable practices.

## Abstract (web summary)

Taking action to reduce risks of labor abuse and illegal, unreported, and unregulated (IUU) fishing in the fishing sector is hindered by a lack of spatially explicit data and an understanding of different drivers of risks. Here the authors combine expert assessments with satellite information to map and quantify risks of labor abuse and IUU fishing at port, at sea and associated with transshipment globally.

## Introduction

Labor abuse and illegal, unreported, and unregulated (IUU) fishing are undermining the social and environmental sustainability of fisheries, with wide-ranging impacts including the loss of individual human rights^1,2^ and nutritional and economic benefits to fishing communities and governments^3–5^. Addressing labor abuse and IUU fishing has been the focus of several recent corporate^6^ and government^7^ policy commitments, but a lack of spatially explicit estimates of labor abuse and IUU fishing risk compromises these actors’ ability to identify and prioritize actions to fulfill them. In spite of their linkages^8–10^, the broad relationships between labor abuse and IUU fishing are only beginning to be explored^11,12^ and the scales and extent of their involvement are not well understood. Although previous research has helped identify risk factors^2,13–15^, there is a need to determine where risks are concentrated and the mechanisms underlying them, to develop the monitoring and enforcement capacity, supply chain transparency, and evidence-based due diligence measures to mitigate risks.

**Fig. 1:**
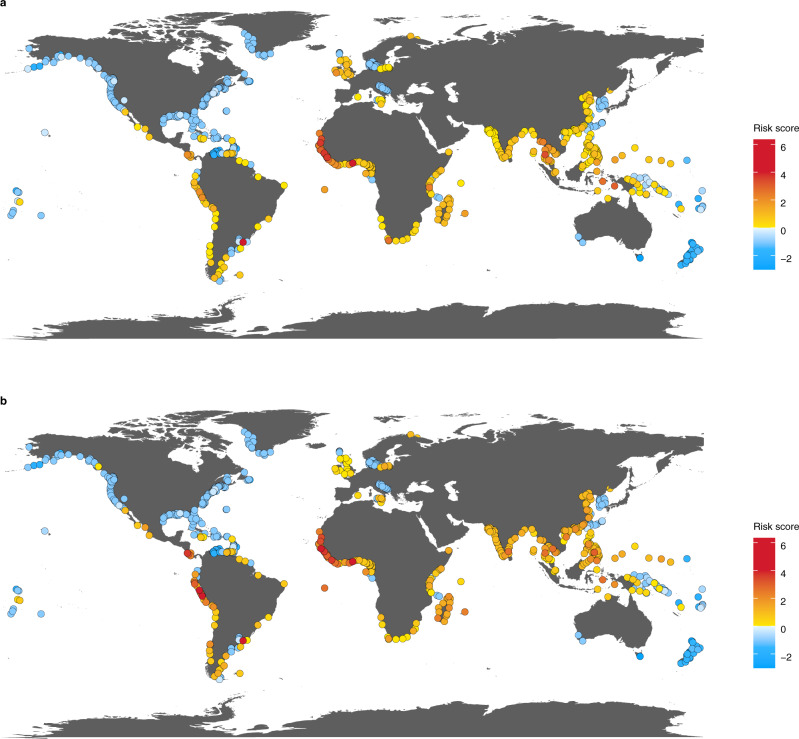
Risk maps for labor abuse and IUU fishing at port. The risk score of ports assessed in the survey for (**a**) labor abuse and (**b**) IUU fishing. Blue indicates lower port risk scores and red denotes higher scores.

To address these gaps, we combined a knowledge co-production expert-elicitation approach^16^ with ‘big data’ from more than 8,768,000 fishing vessel trips and 5800 transshipment carrier vessel trips based on their onboard automatic identification systems (AIS) from 2012 to 2019^17^. We used expert assessments to quantify risks of labor abuse or IUU fishing at ports, globally. Port risks are associated with vessels that may have been engaging in labor abuse or IUU fishing while at sea coming into port (Fig. 1). Labor abuse includes, but is not limited to, subjecting workers to forced labor, moving people into forced labor, poor labor practices, and poor monitoring of labor standards. We focus on labor abuse because it encompasses both egregious violations and other forms of exploitation that may or may not be legally defined. Illegal, unreported, and unregulated fishing is a broad term that includes fishing by national or foreign vessels in contravention of a country’s laws or a Regional Fisheries Management Organization’s conservation and management measures, fishing that is not reported or misreported to the relevant authority, or fishing in areas or for fish stocks to which there are no applicable conservation or management measures^18^. Because most legal frameworks that govern labor abuse and IUU fishing are separate, we consider them independently in this work.

We concentrate on assessing risk, or the likelihood that labor abuse or IUU fishing may be present, rather than occurrence. Prevalence measures are only available for limited geographies, particularly for labor abuse^19^, and remedies need to be in place regardless of case numbers. Therefore, risk can provide a more actionable concept for stakeholders. We pair expert-derived port risk scores for ports where fishing and carrier vessels ended their trips and likely landed catches and exchanged crews (i.e., the arrival port), with fishing and carrier vessel information curated as part of the AIS dataset^17,20^ to identify and characterize higher risk areas associated with fishing and transshipment at sea, separately. Through the modeling framework, we also explore the relative importance of different potential risk predictors (e.g., vessel flags, vessel gear types, etc.) and how risk may be related to actions at port.

**Fig. 2:**
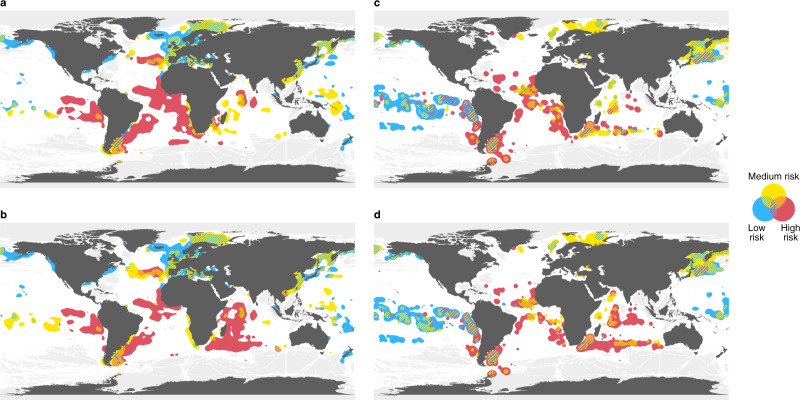
Risk maps for labor abuse and IUU fishing and associated with transshipment at sea. At-sea risk areas based on arrival ports for (**a**) labor abuse and (**b**) IUU fishing, and transshipment risk areas for (**c**) labor abuse and (**d**) IUU fishing. Low-, medium- and high-risk areas are colored in blue, yellow, and red respectively, and overlaps between risk categories are hashed. At-sea areas in white indicate less risk than the colored areas, rather than zero risk. Gray at-sea regions indicate lack of AIS data. For at-sea and transshipment risk areas, each fishing or carrier trip risk score of the arrival port was classified into low-risk (port risk score < 0), medium-risk (0 ≤ port risk score < 2) and high-risk (port risk score ≥ 2), with (**a**, **b**) illustrating areas greater than 95% quantile of fishing cumulative hours by all fishing vessels, and (**c**, **d**) areas greater than 95% quantile of encounter events between carrier vessels and fishing vessels in 2012–2019 for each risk category. For (**a**, **b**), root-mean-square error (RMSE) of cross-validation was 0.88 and Matthews Correlation Coefficient (MCC) was 0.62 for port risk of labor abuse, and RMSE 0.88 and MCC 0.65 for port risk of IUU fishing. For (**c**, **d**), RMSE = 1.14 and MCC = 0.49 on port risk of labor abuse, and RMSE = 1.25 and MCC = 0.45 for port risk of IUU fishing.

**Fig. 3:**
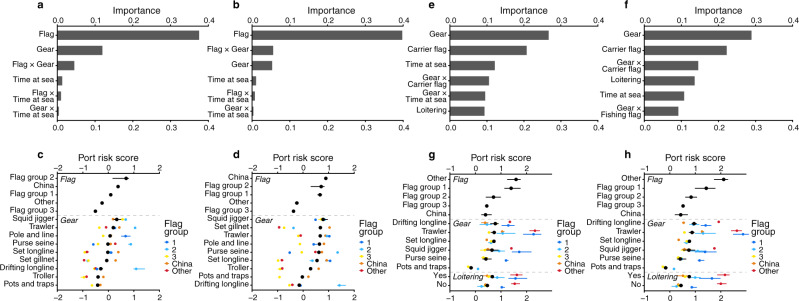
Importance and effects of vessel characteristics in predicting arrival port risks for fishing and carrier (transshipment) vessels. Predictor importance on fishing vessels’ arrival port risk for (**a**) labor abuse and (**b**) IUU fishing, and effects of the predictors for (**c**) labor abuse and (**d**) IUU fishing. Predictor importance on carrier vessels’ arrival port risk for (**e**) labor abuse (top 6) and (**f**) IUU fishing (top 6), and effects of the predictors for (**g**) labor abuse and (**h**) IUU fishing. For (**a**, **b**, **e**, **f**), importance of single predictors was calculated as a mean of absolute SHAP (SHapley Additive exPlanations) values of the individual predictor without accounting for the interaction effects; importance of interactions (represented by x between predictors) indicate additional contribution to the model without accounting for the individual effects of each interacting predictor. Flag groups were classified according to Ford and Wilcox^20^ with China as a separate group, and Flag group 1 corresponding to high ownership by countries other than the flag state, low fidelity to the flag state EEZ and higher control of corruption; Flag group 2 with poor control of corruption, intermediate ownership by countries other than the flag state, and low fidelity to flag state EEZ; Flag group 3 with low ownership by countries other than the flag state, high fidelity to the flag state EEZ, and intermediate control of corruption; and Other as flag states not included in the original cluster analysis (Supplementary Table 2). Black points represent the estimated port risk score when the predictor is present, calculated by adding the main SHAP values (without interaction effects) to the model baseline. For fishing gear type, interactions with flag groups are also shown (flag groups of fishing vessels in (**c**), (**d**); of carrier vessels in (**g**), (**h**)). For (**c**), (**d**), (**g**), (**h**), points and horizontal lines indicate means and 95% ranges of SHAP values, respectively.

Our results illustrate the ubiquity of labor abuse and IUU fishing risks across ports and oceans, with risks often coupled and labor abuse risk more geographically extensive than previously appreciated (Figs. 1, 2). We find that risks are higher for fishing vessel flags primarily associated with poor control of corruption by the flag state^20^ and Chinese-flagged vessels (Fig. 3a–d). Higher IUU fishing risks were also related to flags largely connected with high ownership by countries other than the flag state^20^ (Fig. 3d). For transshipment, higher labor abuse and IUU fishing risks were linked to specific gear types—drifting longliners, trawlers, set longliners, and squid jiggers—as well as carrier vessel flags characterized mainly by high ownership by countries other than the flag state (Fig. 3e–h). We also find that vessels with riskier characteristics may visit countries that have ratified the Port State Measures Agreement (PSMA) less frequently and stop in port for shorter durations. Together, our findings identify where risks are greatest, key risk drivers, and potential pathways for mitigating labor abuse and IUU fishing risks. These results can help eliminate blindspots so that governments can expand enforcement and monitoring capacity where needed and companies can exercise due diligence within seafood supply chains to reduce risks of labor abuse and IUU fishing in global fisheries.

## Results and discussion

### Spatial patterns of risk

Ports were a focus of this analysis as hubs of catch landings^21^ and crew movements^22^, as well as critical junctures for the monitoring and enforcement of the legal frameworks that govern labor and seafood catches^23,24^. Through our survey, experts assessed 792 ports. More than 41% of these ports were associated with labor abuse and 48% with IUU fishing (Fig. 1; Supplementary Table 1). Port risk scores for labor abuse and IUU fishing were highly correlated (*n* = 638, *τ* = 0.897, *p* < 0.001), suggesting that both problems are widespread and linked through activities at port.

For ports that had risk assessments, more than 82% of fishing trips ended in ports associated with labor abuse or IUU fishing. Port risks were not related to cumulative fishing vessel hold capacity or carrier vessel hold capacity, which we used as proxies for port size (*τ* = 0.032, *n* = 39, *p* = 0.789 for labor abuse risk and fishing vessel hold capacity; *τ* = 0.120, *n* = 45, *p* = 0.266 for IUU fishing risk and fishing vessel hold capacity; τ = −0.145, *n* = 39, *p* = 0.233 for labor abuse risk and carrier vessel hold capacity; τ = −0.155, *n* = 45, *p* = 0.171 for IUU fishing risk and carrier vessel hold capacity). More than 85% of trips by all carrier vessels also ended in assessed ports with some degree of risk for labor abuse or IUU fishing. Although port risk scores associated with IUU fishing were similar for carrier and fishing vessels (port risk score of fishing vs. carrier vessels, 0.65 ± 1.03 vs. 0.68 ± 1.41, mean ± standard deviation; Welch’s *t* test, *t* = 1.183, d.f. = 3295.6, *p* = 0.237), carrier vessels visited ports that had disproportionately higher risks than fishing vessels for labor abuse (0.11 ± 1.01 vs. 0.59 ± 1.31; *t* = 20.605, d.f. = 3151, *p* < 0.001). Fishing vessels that had met with carrier vessels disproportionately visited lower risk ports for labor abuse (52% vs. 37%; *χ*^2^_1_ = 99.236, *p* < 0.001) and for IUU fishing (48% with encounters vs. 22% without; *χ*^2^_1_ = 406.060, *p* < 0.001), suggesting that they may have less perceived risk in going to ports that may have more stringent enforcement after they have transshipped.

Our models suggest nearly 9% of fishing effort (hours) was associated with vessels returning to ports that were high-risk for labor abuse or IUU fishing (Fig. 2a, b). We identified at-sea risk areas that have received less global and academic attention, including off the coasts of Peru (Humboldt Current), Argentina and the Falkland Islands (Islas Malvinas), and around the Azores, in addition to more widely discussed locations along the West African coast^4,25^ (Fig. 2a, b; Supplementary Fig. 1). For labor abuse, high-risk areas along much of the west African continent, and the Humboldt Current were larger, and areas in the Western Indian Ocean were smaller (Fig. 2a) than those identified for IUU fishing (Fig. 2b) and limited to areas around the Maldives. We also examined where risk areas would be for labor abuse based on departure ports and found similar patterns in high-risk areas (Supplementary Fig. 2). For IUU fishing, the Western Indian Ocean emerged as a major risk area (Fig. 2b).

Transshipment has long been connected to IUU fishing^26^ and more recently linked to labor abuse^19,27–29^. We defined risky transshipments by whether carrier vessels returned to ports that were associated with either labor abuse or IUU fishing risks. In cases where we did not have port risk scores, we used a modeling approach to infer transshipment risks from the characteristics of the carrier and fishing vessels engaged in the transshipment. For both labor abuse and IUU fishing, we found high-risk areas for transshipment, or places with high concentrations of risky transhipments, off the coasts of Argentina, Peru, Chile and Western Africa, around the Kamchatka peninsula and in the Eastern Tropical Pacific (Fig. 2c, d; Supplementary Fig. 3). For labor abuse, high-risk areas were extensive in the Eastern Tropical Pacific and southwestern Atlantic (Fig. 2c). Distinct high-risk areas associated with IUU fishing emerged off southern Africa reaching nearly across the Indian Ocean to Australia (Fig. 2d).

To test the robustness of the at-sea and transshipment modeling results and to ensure that specific ports were not disproportionately affecting our findings, we randomly dropped a proportion of port risk survey data (10% or 20%) and re-fit the models, finding that predictor importance and the effects on port risk score were not substantially affected (Supplementary Fig. 4; Supplementary Note 1).

### Risk drivers

Understanding the factors that drive risk is important for designing effective policies, collecting relevant data in seafood supply chains, and identifying the stakeholders that need to be involved in reducing risk. Port risk scores were used to determine at-sea risk areas. We found fishing vessel flag to have the greatest impact on predicting port risk for both labor abuse and IUU fishing, followed by vessel gear type^30^ for labor abuse, and the interaction between flag and gear type for IUU fishing (Fig. 3a, b).

We assessed risk associated with vessel flags that were categorized into three groups according to whether fleets were owned primarily by entities from countries other than the flag state, the degree of control of corruption by the flag state, and the degree to which they stayed within the flag state’s Exclusive Economic Zone (EEZ)^20^ (Supplementary Table 2). China was considered as a separate group because of its dominance in fishing effort and number of vessels^17^. In our analysis, Flag group 1 refers to a group of six flag states that had high ownership by countries other than the flag state, higher control of corruption^20^, and low fidelity to the flag state EEZ, meaning they operate primarily outside of the flag state’s EEZ. We refer to this group hereafter as flags having high ownership by countries other than the flag state (Flag group 1; Supplementary Table 1), because that was a dominant characteristic. Flag group 2 is a group of flag states linked to poor control of corruption^20^, intermediate levels of ownership by countries other than the flag state, and low fidelity to the flag state’s EEZ (Flag group 2; Supplementary Table 2), which we refer to as flags having poor control of corruption, because they have the lowest Control of Corruption score among the three flag groups. Poor control of corruption is defined by the Worldwide Governance Indicators as the “…extent to which public power is exercised for private gain, including both petty and grand forms of corruption, as well as “capture” of the state by elites and private interests”^31^. Although all of the flags characterized by high ownership by countries other than the flag state (Flag group 1) are part of the International Transport Workers’ Federation’s ‘Flags of Convenience’, 40% of those in the group with poor control of corruption (Flag group 2) are not (e.g., Bahrain, Kiribati, Madagascar, Portugal, and Samoa). Flags of Convenience are commonly assumed to be associated with labor abuse and IUU fishing^32^ (Supplementary Table 2). Flag group 3 had low ownership by countries other than the flag state, high fidelity to the flag state EEZ, and intermediate control of corruption and the ‘other’ group had 39 flag states that were not evaluated in the original cluster analysis^20^ (Supplementary Table 2).

Results show that flags categorized as having poor control of corruption^20^ (Flag group 2; Supplementary Table 2) and Chinese-flagged vessels, were related to higher port risks for both labor abuse and IUU fishing (Fig. 3c, d). For IUU fishing, risk was also associated with flags that have high ownership by countries other than the flag state^20^ (Flag group 1; Supplementary Table 2). For labor abuse, these patterns were similar for a model based on departure ports (Supplementary Fig. 2). Contrary to a focus on long-duration trips related to labor abuse risk^30^, time-at-sea was less important in predicting at-sea risk (Fig. 3a). Thus, labor abuse may also be prevalent on shorter trips, a finding supported by regional studies^33,34^.

Relationships between vessel gear type and port risk were mediated by fleet usage patterns (Fig. 3c, d; Supplementary Tables 3 and 4). For example, higher risks for IUU fishing for squid jiggers were due to Chinese-flagged vessels using the port of Chimbote, Peru (3%), Callao, Peru (3%), and Montevideo, Uruguay (2%), and Korean-flagged vessels using the port of Montevideo, Uruguay (2%). Similarly, higher risks for labor abuse and trawlers were primarily driven by Chinese-flagged vessels using the port of Yantai, China (21% of trips by trawlers) and Dalian, China (14%), and Uruguayan-flagged vessels using Montevideo, Uruguay (1%).

For transshipment, the gear type of the fishing vessels potentially engaging in transshipment and flag of the carrier vessel were the most important predictors for determining port risk (Fig. 3e, f). Higher risk gear types were drifting longliners, trawlers, set longliners, and squid jiggers. Carrier vessel flags that were higher risk were those associated with high ownership by countries other than the flag state^20^ (Flag group 1; Fig. 3g, h; Supplementary Table 2)^32^, a different set of flags than those that were generally higher risk for IUU fishing. The ‘other’ flag group was also high risk, but had few trips (Supplementary Tables 5, 6). Together, these flag patterns could reflect perceived oversight laxity by the flag state^32^.

**Fig. 4:**
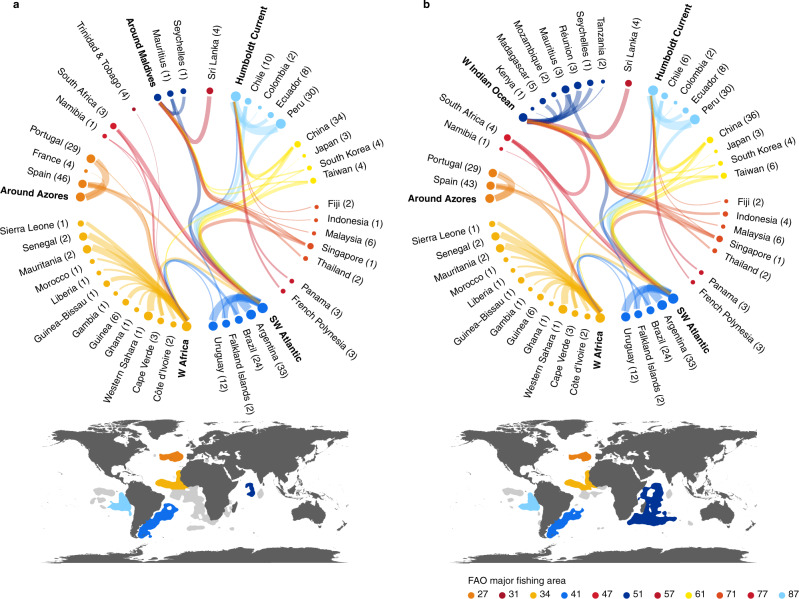
Spatial linkages between at-sea risk regions and ports. Interconnections between selected high at-sea risk regions and arrival port countries for (**a**) labor abuse and (**b**) IUU fishing. Node size and edge width are proportional to the number of trips from at-sea high-risk regions to destination countries (logarithmic scale). Countries within or bordering the same Food and Agriculture Organization (FAO) Major Fishing Area are grouped by color, and correspond to the geographic extent of the at-risk regions in the associated maps. The full extent of the at-risk regions from Fig. 2 are in gray. Text in bold reflect at-sea risk regions displayed in the maps, which were the focal areas evaluated. Values in parentheses are the number of unique destination ports within each country. For simplicity, each figure captures destination countries with more than 10 trips from each at-sea risk area.

Our analyses also underscore the increasingly globalized nature of fishing^35,36^, illustrating not only the prevalence of foreign-flagged vessels in many fishing grounds (Supplementary Tables 7, 8), but also which ports may drive risk in fishing grounds thousands of miles from where catches are landed^21,37^. Although a significant number of vessels ended trips at ports close to high-risk areas (Fig. 4; Supplementary Fig. 5), many also returned directly to (home) ports in Europe, Africa, and Asia (Fig. 4; Supplementary Fig. 5; Supplementary Tables 9, 10). These results highlight previously unappreciated spatial linkages between fishing grounds and the often distant ports where catches are landed, revealing places where greater regional or bilateral coordination may be necessary.

This research was guided by estimates of port risk and available data on fishing and carrier vessel dynamics. Vessel behavior data are predominantly available for the High Seas^17^, so patterns within EEZs may vary. Gaps in AIS data or a lack of port risk assessments may have impacted our ability to identify potential areas of risk for labor abuse and IUU fishing^17,38^ (Supplementary Figs. 6, 7). Our analyses did not distinguish between the various dimensions of IUU fishing (i.e., illegal versus unreported or unregulated) and did not account for the presence of certification or governance structures^39,40^ that may help reduce risk. We have largely focused on potential directions for the private sector to actively engage in cooperative action with other relevant stakeholders, because the lens of our analysis is focused less on end-buyer and consumer leverage and more on better monitoring, control and surveillance at port and at sea. Expanded and more focused due diligence actions by companies and governments will likely continue to be necessary because of enforcement challenges and an absence of effective auditing systems for labor abuse^41^.

### Opportunities for greater stewardship

**Fig. 5:**
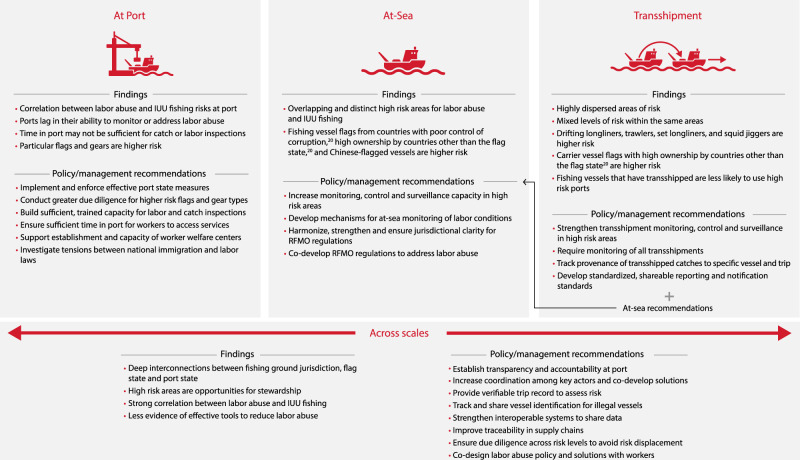
Conceptual framework linking results to key recommendations for mitigating risks of labor abuse and IUU fishing at port, at sea, and with transshipment. Key actors, including governments, companies, Regional Fisheries Management Organizations, and civil society organizations, will need to actively coordinate and consult with each other on most of the proposed recommendations. Certain actors may need to play lead roles in particular recommendations. For example, governments and Regional Fisheries Management Organizations could lead on strengthening data sharing systems and creating enforcement strategies based on risk, companies on fostering supply chain transparency and traceability, and civil society groups on helping to ensure representation across stakeholders.

Our results highlight several pathways that private and public sector actors can take to reduce risks at port and at sea (Fig. 5). Establishing greater transparency through sharing vessel tracks can establish catch and port provenance, which could help governments and companies assess risk and guide resources more effectively. Coordination across flag states, Regional Fisheries Management Organizations (RFMOs) and port states could help identify risky vessels more effectively, and allocate additional monitoring and enforcement capacity in high-risk areas accordingly. At the same time, data collection and due diligence actions as part of procurement procedures within seafood supply chains could help ensure capacity at higher risk landing ports, vessel flags and gear types (Fig. 5). Maintaining and transferring information on points of risk throughout global supply chains, potentially including market-based instruments, where appropriate, may support transparency and improve accountability.

Our analyses suggest that ports may be an important fulcrum for reducing both labor abuse and IUU fishing. To evaluate how measures at port could reduce IUU fishing, we focused on analyzing the impacts of Port State Measures Agreement (PSMA) ratification and entry into force in 2016 on vessel behavior. PSMA is designed to reduce IUU fishing by making it difficult for foreign-flagged fishing and support vessels (e.g., bunker and carrier vessels) to land or transship IUU catches and obtain supplies^42^, by stipulating more rigorous procedures at port, including notification and inspection, data exchange so that authorities are aware of whether a vessel has violated regulations in other jurisdictions, and port access denial, if necessary^43^.

**Fig. 6:**
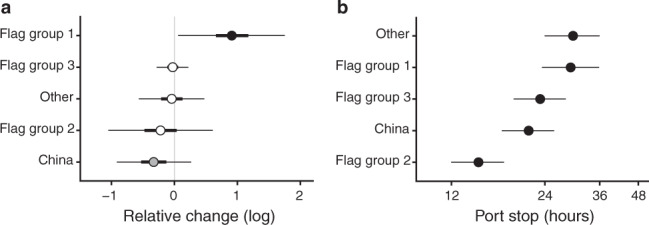
Vessel behavior related to Port State Measures Agreement (PSMA) entry into force and port duration. (**a**) Relative changes in the number of visits by foreign vessels to PSMA parties by flag group for fishing vessels between 2015 (pre-PSMA) and 2017 (post entry into force). Points are medians of Bayesian posteriors, and thin and thick horizontal lines are 95% and 50% credible intervals (CIs), respectively. Vertical line at 0 indicates no change. Black points indicate 95% CIs that do not overlap with zero, gray points indicate 50% CIs do not overlap with zero, and white points indicate overlap. (**b**) Port stop duration by flag group for fishing vessels, using gear type and port state as random effects. Points and horizontal lines represent the best estimates and 95% confidence intervals, respectively. See Fig. 3 for flag group descriptions and Supplementary Table 2 for countries associated with different flag groups.

Certain fishing vessel flags, including China (–28% [95% credible intervals: –60, +31]) and those categorized as having poor control of corruption (Flag group 2; –20% [–65, +84]) (Fig. 6a), as well as certain gear types (Supplementary Fig. 8a), were less likely to visit countries that had ratified PSMA one year after PSMA entered into force, compared to the year before PSMA ratification, although uncertainty estimations were large. Notably, however, these fishing vessel flags were the same as those that we found to be associated with higher risks of labor abuse and IUU fishing (Fig. 3c, d). PSMA ratification and entry into force may have caused a perceived increase in the likelihood of detection of IUU catches and could be an important tool in efforts to reduce IUU fishing, although further analyses will be needed to determine whether these patterns persist over time.

Declines in the likelihood of port visits to countries that have ratified PSMA after entry into force also point to the need for global ratification and effective implementation of this agreement or similar port state measures by all countries. PSMA is predicated on the assumption that if enough countries implement and enforce stringent port state measures, landing IUU catches by foreign fleets becomes more challenging and costly. Ratification across neighboring countries should reduce risk displacement, given the importance of fuel costs in determining profitability^44^. Patterns may shift if vessels perceive that stronger port state measures are not actually in place. Effective implementation will require adequate capacity and training for personnel and coordination among relevant national entities associated with monitoring, control, and surveillance, as well as strengthening the infrastructure for data sharing that can enable them to monitor activities across jurisdictions (Fig. 5). These actions will need to be consistent across space and over time and potentially extend beyond current regional bodies, given the global movement of vessels (Fig. 4). The establishment of legal frameworks that support authorities to implement and enforce regulations will also be necessary (Fig. 5). Consistent, effective regional implementation reduces the possibility of a ‘race to the bottom’ to the least well-regulated countries or ports.

The PSMA is not designed to reduce labor abuse, but other measures at port have potential to address these issues. Broadly, there is a paucity of evidence-based assessments of interventions for labor abuse. Nonetheless, an opportunity to identify and respond to labor abuse has shown promise in mitigating risks in other contexts^45,46^. One of the few places to identify and respond to labor abuse is in port^47^, which depends on the vessel’s port duration or time in port, the ability of fishers to disembark^48^, the capacity of port inspectors^47^, and fishers’ access to port services where labor conditions could be reported. Inability to disembark because of short port durations or immigration restrictions are recognized to exacerbate labor abuse, and have driven recent legislative changes^49^. Port durations varied by vessel flag (Fig. 6b) and by vessel gear type (Supplementary Fig. 8b), but may not be of sufficient duration for identifying abuse through quality inspections or responding to it through port services, given the complexities of disembarkation^50^. For labor abuse, these challenges can be particularly acute for vessels flagged to states with poor control of corruption (Flag group 2; Supplementary Table 2), which spent shorter times in port (${\chi }_{4}^{2}$ = 923.162, *p* < 0.001; Fig. 6b), and therefore may have had fewer opportunities for inspections.

Our results find risks even in places where laws and policies that aim to reduce ‘modern slavery’ or forced labor are in place^12,51^, suggesting there may still be a wide gulf between intention and implementation, a finding consistent with human trafficking more broadly^52^. Exemptions for fisheries within national labor laws^29^ or conflicts with immigration laws that can impede implementation of labor laws^53,54^ complicate enforcement. A range of actions will be needed to mitigate labor abuse, including closing legal loopholes in national regulatory frameworks that govern labor conditions on fishing vessels and developing more effective monitoring and reporting mechanisms that are co-designed with workers and worker rights organizations (Fig. 5).

Although market-based solutions are common in agricultural supply chains, evidence suggests that labor abuse persists in many of them. For example, similar to the seafood sector, cocoa and tea have voluntary multi-stakeholder initiatives designed to combat forced labor. However, recent work has found that forced labor prevalence was not lower in plantations that were ‘fair trade’ certified^55,56^. Implementation of voluntary market-based solutions that are not anchored to binding commitments (market-based or regulatory)^57^ have been less effective for labor abuse in supply chains where it is systemic,^55^ and where rising production costs, highly competitive sourcing, and relatively flat or declining prices are prevalent^55,56^, characterisics present in the seafood sector^2^. Stronger worker-driven verification systems and human rights due diligence would need to be in place before market-based solutions like ethical certifications should be considered^55^. In the interim, greater scrutiny is needed for higher risk vessels (Fig. 3c) and of policies that currently limit port access to workers and hinder reporting of abuse. There are efforts underway to incorporate market-based solutions as part of transformative worker-driven social responsibility. Such an approach relies on protections being worker-driven, enforceable, and subject to legally binding commitments for companies, a combination that has not yet come together in the seafood sector.

## Conclusion

Co-located risks at ports and similar risk drivers for labor abuse and IUU fishing offer opportunities for synergistic action across the range of activities needed to mitigate them. For example, across independent analyses for labor abuse and IUU fishing risks, flag states with poor control of corruption (Flag group 2; Supplementary Table 2) and Chinese-flagged fishing vessels were found to be related to higher risks for labor abuse and IUU fishing (Fig. 3c, d), reduced vessel visits to PSMA-ratified countries (Fig. 6a), and shorter port durations (Fig. 6b), suggesting a need for greater focus on these vessels in port controls and inspections. Additional due diligence measures in company supply chains for vessels with these characteristics could also be prioritized, particularly in high-risk ports^20,47^ (Figs. 1, 5). Similar actions may also be needed for carrier vessels flying flags that are associated with high ownership by countries other than the flag state (Flag group 1; Supplementary Table 2), and higher risk fishing vessel gear types that may have transshipped (Fig. 3g, h). Although the specific actions needed for reducing labor abuse and IUU fishing may be different (Fig. 5), establishing transparency and accountability at port holds promise in reducing risks for both.

Higher risk areas are challenges for governments, seafood companies, and other key actors to manage collaboratively. Remedies need to focus on addressing risk, rather than moving operations elsewhere, which would only displace risk and may be operationally difficult due to fuel efficiency and logistics^58,59^. Instead, high-risk areas offer opportunities for greater stewardship by actors to reduce risk in places where they operate or have jurisdiction. Increased coordination can leverage the particular strengths of each actor, including enforcement and monitoring capacity, market-based or financial incentives, social norms and governance control, to focus on places our results suggest are at greatest risk. Previous work in fisheries has illustrated that collective action between diverse actors generates results^60,61^. By wielding their respective powers, key actors can take concerted action across supply chains, create or improve regulatory frameworks, and catalyze change in industry behavior to reduce the risks of labor abuse and IUU fishing in global fisheries.

## Methods

This research complies with all relevant ethical regulations. The study protocol was evaluated and approved by the Stanford Institutional Review Board (Stanford IRB #49308).

### Port risk assessment

Port risks associated with labor abuse and IUU fishing were evaluated through an online global survey in English, Spanish and French, deployed using the platform Qualtrics. Within the survey instrument, all participants were provided with information about the study and advised that their participation was entirely voluntary and anonymous (i.e. that responses could not be tracked to an individual). They were also advised how they could contact Stanford IRB with any concerns. Informed consent was documented by tick-box to ensure anonymity, because signature would have provided identifiable information. Participants were also advised of withdrawal procedures, and that they could opt out of taking the survey at any time by closing their browser.

Over 95 experts from seafood companies, research institutions, human rights organizations, and governments addressed questions in the survey that evaluated the risk levels for labor abuse and IUU fishing associated with ports, and answered questions about other known risk factors including vessel flag^20,32,62^, vessel gear type^19,63^, and time-at-sea^22^. Based on respondents’ selected countries of expertise, the survey provided a list of ports for those countries. For each port, respondents were instructed to indicate whether, based on their experience, the port was known for labor abuse or IUU fishing, and if so, their level of certainty about that association. Within the survey, we defined *low certainty* as the port being known to be poorly monitored or regulated, *medium certainty* as the port having suspected occurences of labor abuse or IUU fishing, and *high certainty* as the port having documented cases of labor abuse or IUU fishing. When the port was not associated with risk of either IUU fishing or labor abuse, respondents were asked to select *not associated*.

### Port risk scores

To describe fishing grounds associated with risk of labor abuse and IUU fishing, we focused on the ports used by fishing vessels to likely land their catches (i.e., the arrival port). For each port, survey responses were converted to a value of −1 for *not associated*, and 1/3, 2/3 and 1 for *low*, *medium* and *high certainty* of association with labor abuse or IUU fishing, respectively. We assigned each fishing trip a port risk score by summing all responses for each arrival port (Supplementary Fig. 9), although we also explored departure port risk for labor abuse because movement of people into situations of labor abuse could be related to conditions at the departure port (Supplementary Fig. 2). Consequently, port risk scores took into account both the number of responses and the level of agreement among the respondents on their level of certainty for each port (Supplementary Fig. 9). Low scores indicate that several respondents evaluated the ports used during the fishing trip as not associated with labor abuse or IUU fishing risks, whereas high scores indicate high certainty among multiple respondents that the port is exposed to high risks of labor abuse or IUU fishing. We removed all data where respondents indicated two different levels of certainty when assessing one dimension of risk, e.g. selected both *not associated* and *low* for IUU fishing.

### Modeling approach

The port risk score of the fishing trip was then linked to information about vessel movement and fishing activity from Global Fishing Watch (GFW) to model at-sea and transshipment risks. Data were extracted from GFW databases on September 15, 2021, and reflect updates to that date. From the onboard Automatic Identification System (AIS) data, GFW identified over 8.7 million fishing trips from 2012 to 2019 that include information on the vessel flag and fishing gear type. GFW also determines when fishing occurs on a trip and estimates the number fishing hours at each observed location through machine learning based on vessel movement patterns^64^. We matched the end coordinates of the fishing trips reported by GFW with the nearest port in our survey by searching within a 3-km radius from each port. We were then able to assign port risk scores for 1.8 million trips based on 412 ports for the labor abuse analysis and 3.0 million fishing trips based on 455 ports for the IUU fishing analysis.

Using these data, we developed a model to identify traits of fishing vessels that contribute to port risk. Predictors used in the model were flag groups (5 levels: Flag group 1, Flag group 2, Flag group 3, China, Other) (Supplementary Table 2), gear types (9 levels: set longline, drifting longline, squid jigger, pots and traps, trawlers, set gillnet, pole and line, trollers, purse seine), and time-at-sea (5 levels: less than 1 month, 1–3 months, 3–6 months, 6–12 months, 12 months and more). Categorization of gears is based on available data for training the models that GFW uses in their classification. Gears for which less data were available may have greater classification error.

These predictors were chosen because they represent known risk factors for labor abuse or IUU fishing^19,20,22,32,62,63^ and were vessel characteristics that were standardized and consistent within the GFW database. We categorized vessel flag groups based on Ford and Wilcox^20^, who used globally consistent indicators of foreign ownership (ratio of nationally flagged to nationally owned vessels), control of corruption, and fidelity to the flag state’s exclusive economic zone (EEZ), including territorial and archipelagic waters (i.e. remaining within the flag state’s EEZ), to better understand what aspects of vessel flag may be driving risk (Supplementary Table 2). Flag group 1 (6 states for this study) is characterized by a high ownership by countries other than the flag state (high foreign ownership ratio), high proportion of vessels operating outside their EEZ (Supplementary Table 2) and higher control of corruption. Flag group 2 (20 states for this study) is mainly distinguished by poor control of corruption, as well as low fidelity to the flag state EEZ, and intermediate levels of ownership by countries other than the flag state (Supplementary Table 2). Flag group 3 (91 states for this study) represents flag states with high ownership by the flag state, a high proportion of vessels operating within their flag state EEZ, and intermediate control of corruption (Supplementary Table 2). Although China was clustered in Flag group 3 by Ford and Wilcox^20^, we separated China as its own flag group because of its dominance in the data (41% of 8.7 million fishing trips with all predictors), which could otherwise mask the predictive power of other flag groups. The flags that did not fall into the above three categories were grouped as ‘Other’ (39 states for this study) (Supplementary Table 2).

### Port risk and cumulative fishing vessel and carrier hold capacities by port

To determine whether experts simply perceived port risk as a reflection of port size, we investigated the relationship between port risk score and cumulative fishing and carrier vessel hold capacity estimates for major ports^65^ (*n* = 99), as proxies for port size. We matched these ports with our data (39 ports for labor abuse risk and 45 ports for IUU fishing risk) and assigned port risk scores from our survey. We tested the correlation between port risk score and cumulative fishing vessel hold capacity and separately, for port risk score and carrier vessel hold capacity, using Kendall’s rank correlation.

### At-sea risk model

To estimate at-sea risk, we created decision trees for labor abuse and IUU fishing, each trained with over 100 trees with gradient boosting^66^, with a maximum depth of 10, shrinkage of 0.05, a predictor subsampling rate of 0.6, and a root-mean-square error (RMSE) as a loss function. We evaluated the importance and effect of each predictor to the model through SHAP (SHapley Additive exPlanations) values^30^. Grounded in cooperative game theory^67^, the SHAP value measures the contribution of each predictor to the prediction with respect to a model baseline. In our case, it measures a change in the predicted port risk score attributed to each predictor for each fishing trip. Predictor importance was calculated as ${I}_{j}=\frac{1}{n}\mathop{\sum }\nolimits_{i=1}^{n}|{\phi }_{j}^{i}|$, where *j* is the predictor, *n* is the number of observations, and ${\phi }_{j}^{i}$ is the SHAP value of predictor *j* in observation *i*. Larger values indicate greater contribution to the model prediction. For each predictor with high contribution to the model, we estimated the main effect to the predicted port risk score by computing SHAP values when the predictor was present in the trip. We obtained the predicted port risk score solely attributed to the predictor by adding the main effect to the model baseline without accounting for interaction effects. To explore the potential interactions between predictors of interest, we also obtained the predicted port risk score by adding the main effect and the interactions to the model baseline. The model was fitted using ‘XGBoost’ 1.0.0 under Python 3.7.1. SHAP values were computed using ‘shap’ 0.35.0 under Python 3.7.1.

We summarized spatial risk at-sea based on arrival port risk score. Based on the distribution of the observed risk score of both labor abuse and IUU fishing, port risk score was binned into three groups (low: risk score < 0, medium: 0 ≤ risk score < 2, high: risk score ≥ 2) using a univariate k-means method^68^ (Supplementary Fig. 9). Subsequently, each fishing trip was categorized into one of three classes based on the expert-assessed port risk group of the arrival ports. For fishing trips that did not have an expert-assessed port risk (7.0 million trips for labor abuse, 5.8 million trips for IUU fishing), we predicted the port risk score using the model and categorized these trips into one of three classes in the same way. For each class of fishing trips (observed and predicted combined), we obtained cumulative fishing hours over all fishing vessels in a 1 × 1-degree grid. Fishing hours were then scaled to the corresponding areas in km^2^. The maps identified the top 5% of values and were created using bivariate Gaussian kernel density estimation, weighted by the scaled cumulative number of fishing hours in each grid, with a band width of five degrees.

### Transshipment risk model

Transshipment risk areas were modeled using trips taken by carrier vessels that had encounters with fishing vessels during the course of their trip. We also used single-vessel loitering events^69^ as a predictor in the model. Two-vessel encounters are defined as two vessels remaining within 500 m of each other for longer than 2 h, traveling at less than 2 knots while at least 10 km from an anchorage^69,70^. Single-vessel loitering events occurred when carrier vessels stayed at least 20 nautical miles from shore, traveling at less than 2 knots for 8 h or more, vessel behavior consistent with transshipment, but with no other vessel observed through AIS in the immediate vicinity^69^. Loitering can be indicative of transshipment with a vessel that has an AIS transponder that has been turned off or the transshipment vessel waiting until its next task. Under these criteria, GFW listed 5,811 trips by carrier vessels that encountered at least one fishing vessel from 2012 to 2019. By matching AIS information of these trips with coordinates of expert-assessed ports in our survey, we assigned port risk scores to 3,229 trips based on 76 ports for labor abuse risk and 3,386 trips by carrier vessels based on 82 ports for IUU fishing risk.

Using these data, we developed decision tree models for labor abuse and IUU fishing to identify areas of higher transshipment risk by estimating arrival port risk of trips by carrier vessels. The models were each trained with over 300 trees with gradient boosting, with a maximum depth of 10, shrinkage of 0.05, a subsampling rate of 0.6, and RMSE as a loss function. Predictors in the model were flag type of a carrier vessel, time-at-sea, flag type of encountered fishing vessels and their gear types, and the occurrence of a loitering event. Flag type (of both the transshipping fishing vessel and carrier vessel), gear type of the transshipping fishing vessel, and time-at-sea were categorized in the same way as in the model of at-sea risk and converted to numeric with one hot encoding. Predictor importance was evaluated using SHAP values.

We visualized spatial risk of transshipment using port risk score in a similar way to at-sea risk. Specifically, we assigned the port risk class of the trip to the coordinates of an encounter event during the trip. When trips by carrier vessels did not have an expert-assessed port risk (2,582 trips for labor abuse and 2,425 trips for IUU fishing), we predicted port risk score using the model and categorized these trips into one of three classes. For each class, we summed the number of all encounter events in a 1 × 1-degree grid and scaled these to the corresponding areas in km^2^ and presented the top 5% of values. The maps were created using bivariate Gaussian kernel density estimation, weighted by the scaled density of transshipment encounter events in each grid, with a band width of five degrees.

### Model performance and robustness analyses

We evaluated the performance of our models using root-mean-square error (RMSE) with 10 × 5-fold cross-validations (Fig. 2). We also evaluated model performance using Matthews Correlation Coefficient (MCC) by 10 × 5-fold cross-validations after categorizing predicted port risk score into three risk classes (Fig. 2).

To evaluate the robustness of our modeling results, we simulated cases where we had received fewer responses from experts in the port risk survey by randomly dropping 10% or 20% of the experts’ responses from the survey and re-assigning a port risk score to each port. Using the subsampled data, we re-fit the model with the same hyperparameters and calculated predictor importance and effects on port risk score through SHAP values. The procedure was repeated 10 times for 10% and 20% drops (Supplementary Fig. 4; Supplementary Note 1). The similarity of predictor importance and effects among subsamples was measured as intraclass correlation (ICC), and agreement was tested using *F*-test (Supplementary Note 1). We also assessed agreement among experts using Shannon entropy and found that it was medium to high among experts assessing risk at port (Supplementary Fig. 9), indicating that our survey captured shared perceptions of port risk.

### Port stop duration

We obtained the duration of port stops by fishing vessels using the database curated by GFW. From AIS signals, GFW records the location and time of three events when vessels are at port: (1) the beginning of a port stop event, defined as when a vessel travels at less than 0.2 knots while in port (within 4 km of an anchorage point), (2) the ending of a port stop event, defined as when a vessel travels at greater than 0.5 knots while in port, and (3) port gap, when a gap between AIS signals is greater than four hours while in port. We calculated the duration of a port stop as the time difference between the beginning and ending of the port stop event that took place at dock. We removed port stop events where the beginning of a port stop event did not immediately follow the port entry or the ending of the previous port stop, or where the ending of a port stop event was not immediately followed by port exit or the beginning of the next port stop. We merged two consecutive port stops when they took place at the same anchorage point and the gap was less than 30 minutes. We also removed port stops of less than one hour, which may be vessels moving slowly at or between anchorage points. After removing potentially spurious events, we identified 6 million vessel port stop events between 2012 and 2019, among which were over 183,000 visits by foreign vessels that we used in the analysis.

We then compared port stop duration of foreign fishing vessels across flag groups. In a linear mixed-effects model, we specified logarithm of port stop duration (hours) as a response variable and flag group as an explanatory variable. We also included port state and fishing gear type as random effects to account for potential differences in port infrastructure between countries and known differences in unloading and fueling times for different vessel gear types due to the vessel and catch size. We used a similar approach to model port stop duration by gear type using port states as a random effect. To be consistent with the risk mapping, we limited the analysis to the vessels with specified flag states and gear types in the GFW database. The model was run using ‘lme4’ ver. 1.1–26^71^ in R 4.0.4, and the confidence intervals were estimated using 1000 bootstraps.

### Risk relationships

To understand correlations between labor abuse and IUU fishing risks, we used Kendall’s rank correlation. We hypothesized that risk may change with transshipment, so we explored whether there were differences in the risk of ports that fishing vessels visited, between those that did and did not transship using *χ*^2^ test of independence.

### Regional differences

To explore regional differences in fishing patterns in high-risk areas, we selected 6 high-risk area subsets for labor abuse and IUU fishing: Southwest Atlantic, Humboldt Current, around the Maldives (for labor abuse) or the Western Indian Ocean (for IUU fishing), West Africa, around the Azores, and around the Galapagos Islands (Supplementary Fig. 5). We identified the proportion of flag states and gear types in these high-risk regions for both labor abuse and IUU fishing based on fishing hours (Supplementary Tables 7, 8, 11, and 12). We also identified the top destination ports in the identified high-risk regions based on number of trips (Supplementary Tables 9 and 10).

### Port State Measures analysis

The Port State Measures Agreement (PSMA) focuses on due diligence around foreign-flagged fishing and support vessels (e.g., carrier and bunker vessels). Territories often maintain separate flag identities from their sovereign states (e.g., Greenland and Denmark, Anguilla and the United Kingdom, etc.). For this analysis, we consider these flags to be ‘domestic’ with respect to the sovereign state, because many sovereign states enter into PSMA on behalf of their territories^72^. As ‘domestic’ vessels, they are not subject to the additional measures stipulated by PSMA. Because of the size of the fleets involved, and the legal status of Chinese Taipei with respect to PSMA, we treat vessels flagged to the fishing entity of Taiwan and Chinese-flagged vessels as separate entities^13^ (10.5% of visits flagged to the fishing entity of Taiwan were observed in mainland China, and 0.2% of visits by Chinese-flagged vessels were observed in the Chinese Taipei EEZ in 2012–2019).

To evaluate the initial impact of PSMA ratification and entry into force in 2016 on vessel dynamics, we analyzed how the number of vessel visits to each port state changed between 2015 (pre-PSMA) and 2017 (post entry into force) by 65 port states^72^. In the analysis, we used port states that constitute the top 95% of the cumulative number of port visits to remove port states that received few visits by foreign fishing vessels for each flag group. We also excluded port states that ratified PSMA in 2017 from the analysis to keep the calendar comparisons consistent (i.e., Albania, Denmark on behalf of Greenland and the Faroe Islands, Djibouti, Japan, Kenya, Madagascar, Maldives, Mauritania, Montenegro, Namibia, Peru, Senegal, Togo). For each port state, we counted the number of visits by ‘foreign’ fishing vessels (i.e., vessels flagged to states other than the port state or its territories) for each flag group. For consistency, we limited the analysis to the vessels we used in the risk mapping analysis, and to vessels that were found to be active both in 2015 and 2017 in the GFW database.

We performed Bayesian hierarchical models under the before-after-control-impact design with a log-normal error distribution. In each model, we specified the number of port visits by foreign vessels as a response variable, sampling year (2 levels: 2015 and 2017), PSMA ratification in 2016 (2 levels: yes or no) and the interaction as explanatory variables, and port state as a random intercept. The response variable was linearly scaled by dividing by the maximum value in each model. The coefficient of the interaction indicates the proportional change of visits by foreign vessels to PSMA ratifiers between 2015 and 2017, after controlling for the change in non-PSMA ratifiers. For priors, we used a normal distribution with *μ* = 0 and *σ* = 10 for the mean of each fixed effect and a half-Cauchy distribution with *β* = 5 for the error term. For the random intercept, we used a normal distribution with *μ* = 0 and *σ* specified as a half-Cauchy distribution with *β* = 5. We sampled 2 chains of 5000 samples with 2000 burn-ins each. The analysis was performed using PyMC3 ver. 3.11.2^73^ in Python 3.7.8.

### Reporting summary

Further information on research design is available in the Nature Research Reporting Summary linked to this article.

## Supplementary information

Supplementary Information

Reporting Summary

## Data Availability

Due to the sensitive nature of the port data, the specificity of the geographic information, and the associated ethics considerations, raw port data cannot be made publicly available. Requests for access to the data may be negotiated through a data use agreement and will need to be consistent with the ethics protocol and conditions of informed consent. Please contact Elizabeth Selig (eselig@stanford.edu) for more details. The AIS vessel location data that support the findings of this study are available from Global Fishing Watch https://globalfishingwatch.org/. All other data that were used to produce the results, including ratification dates of PSMA and C188 as well as fishing vessel and carrier vessel hold capacity data are available at: 10.5281/zenodo.5775311.

## Code availability

All code used to generate the results, figures, and tables in this study are publicly available at 10.5281/zenodo.5775311.
