# Supplementary material for: Revealing global risks of labor abuse and illegal, unreported, and unregulated fishing
Source: Nat Commun. 2022 Apr 5;13:1612. doi: 10.1038/s41467-022-28916-2 (PMC8983709; doi:10.1038/s41467-022-28916-2)
Supplement: Supplementary file 1 — Supplementary Information [file 41467_2022_28916_MOESM1_ESM.pdf]

Supplementary Information

Supplementary Figures

Supplementary Tables

Supplementary Note

Supplementary References

Supplementary Figures

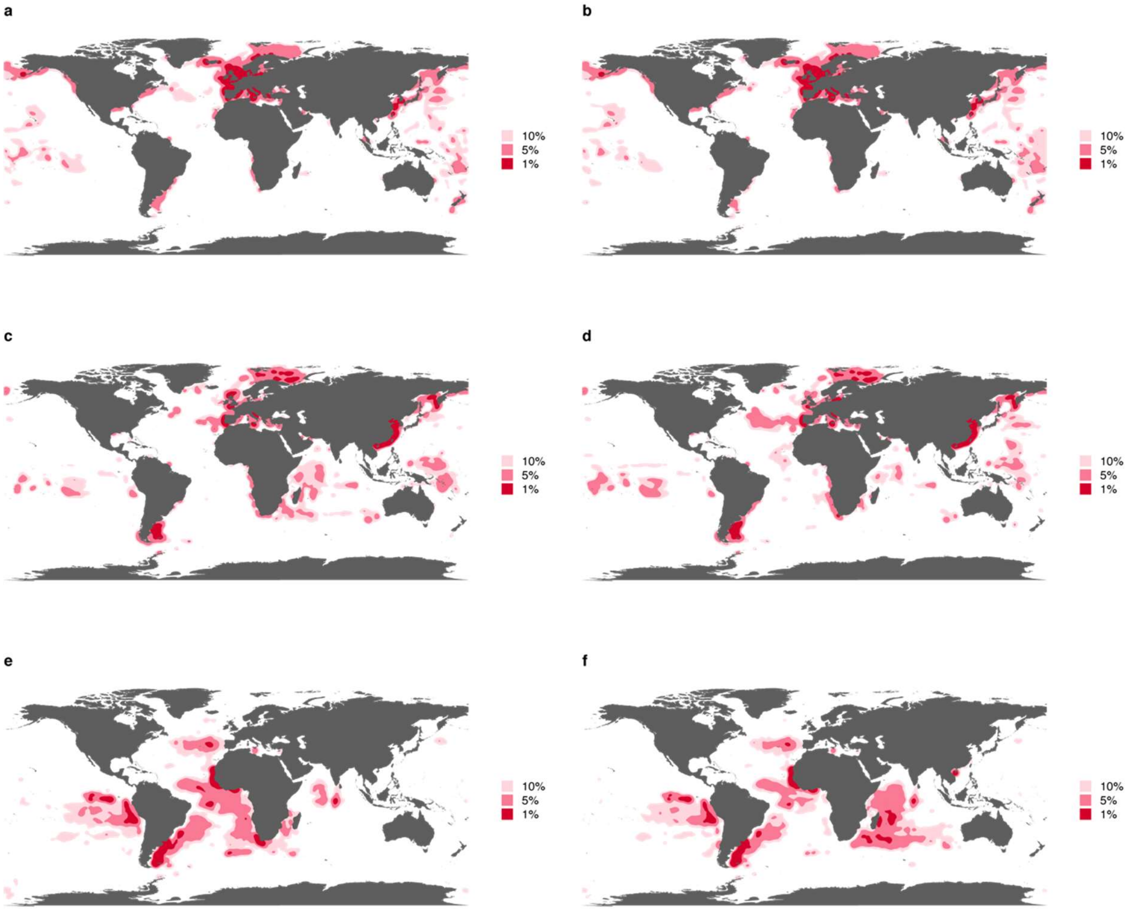

Supplementary Figure 1. At-sea risk areas for each port risk class. Risk regions are based on relative fishing effort and observed and predicted port risk according to flag group, gear type, and time-at-sea for fishing vessel trips ending at low-risk ports for (**a**) labor abuse and (**b**) IUU fishing, medium-risk ports for (**c**) labor abuse and (**d**) IUU fishing, and high-risk ports for (**e**) labor abuse and (**f**) IUU fishing. Colors indicate the quantiles of cumulative fishing hours within each risk class (10% = 90% quantile, 5% = 95% quantile, 1% = 99% quantile). Matthews correlation coefficients for classification were 0.62 for labor abuse fishing and 0.65 for IUU fishing.

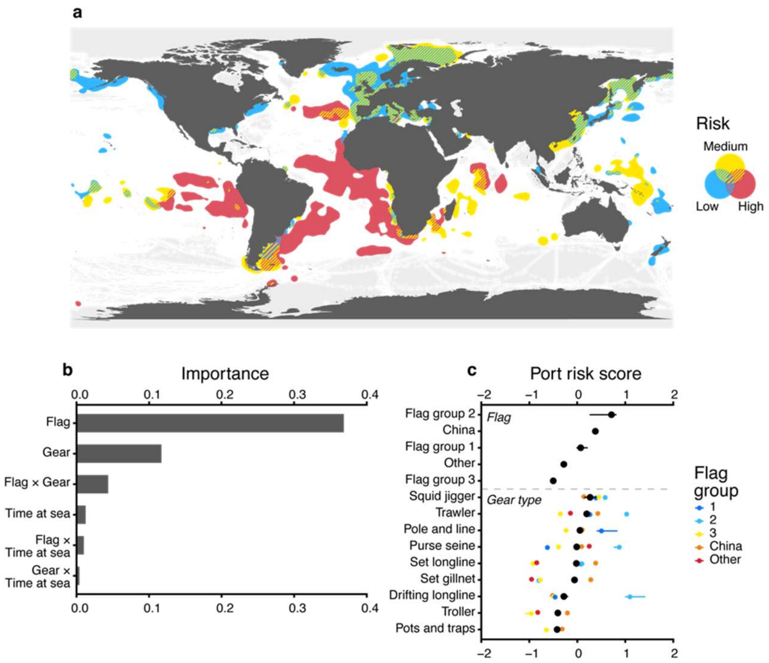

Supplementary Figure 2. At-sea results for labor abuse using departure port risk score. (**a**) The model with the same structure as the one described for arrival port was re-trained using port risk score at departure port. Low-, medium-, and high-risk areas are colored in blue, yellow and red, respectively, and overlaps between risk categories are hashed. Grey at-sea regions indicate no data. We obtained a root-mean-square error 0.88 and Matthews correlation coefficient 0.62 based on 10 × 5-fold cross validation. (**b**) Variable importance on fishing vessels' departure port risk for labor abuse. Interactions are represented by 'x' between variables. (**c**) Effects of each variable on port risk of labor abuse. Black points present the estimated port risk score when the variable is present, calculated by adding the main SHAP (SHapley Additive exPlanations) values (without interaction effects) to the model base line. Numbers in the top flag category represent flag classes as categorized in Supplementary Table 2 based on Ford and Wilcox[1]. For fishing gear type, interactions with flag groups are also shown. For **c**, points and horizontal lines indicate means and 95% ranges of SHAP values, respectively.

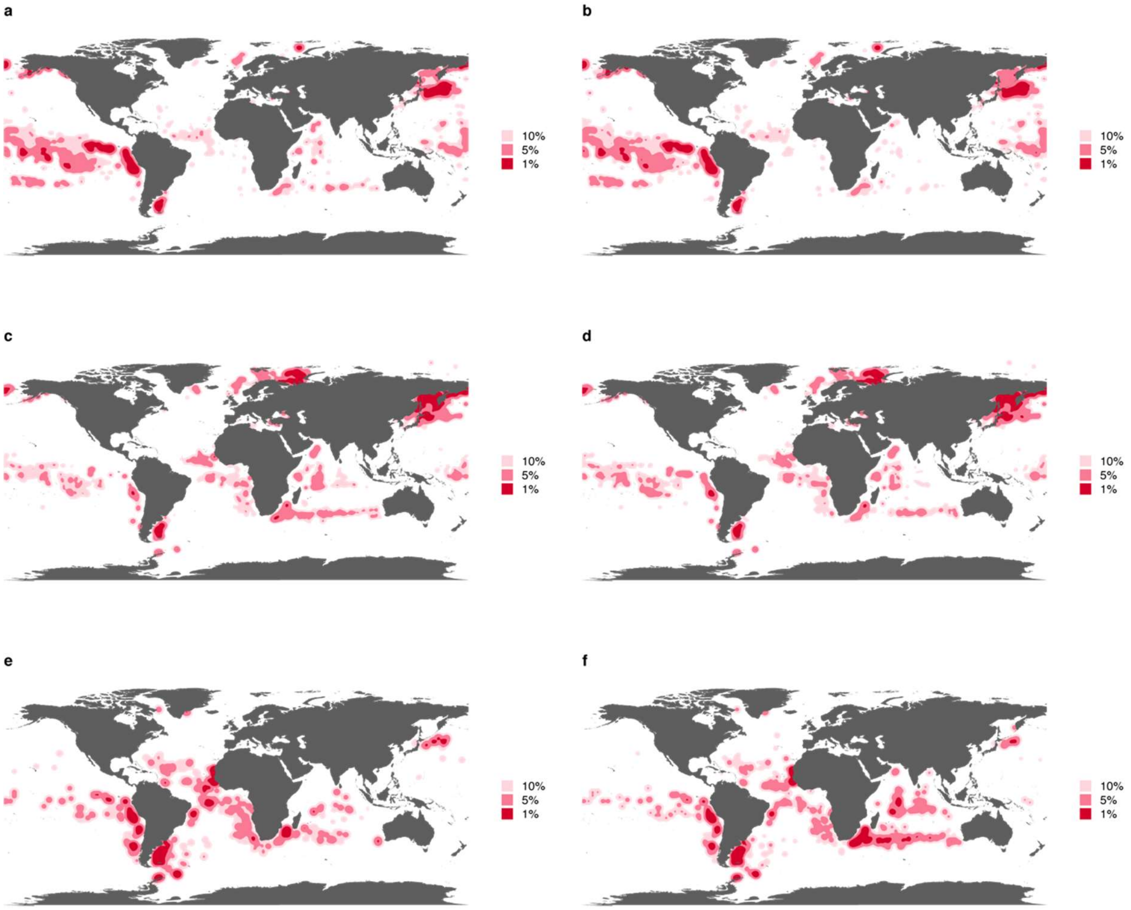

Supplementary Figure 3. Transshipment risk areas for each port risk class. Risk regions are based on occurrence of encounter events and observed and predicted port risk by carrier vessels arriving at low-risk ports for (**a**) labor abuse and (**b**) IUU fishing, medium-risk ports for (**c**) labor abuse and (**d**) IUU fishing, and high-risk ports for (**e**) labor abuse and (**f**) IUU fishing. Colors indicate the quantiles of cumulative fishing hours within each risk class (10% = 90% quantile, 5% = 95% quantile, 1% = 99% quantile). Matthews correlation coefficients for classification were 0.49 for labor abuse and 0.45 for IUU fishing.

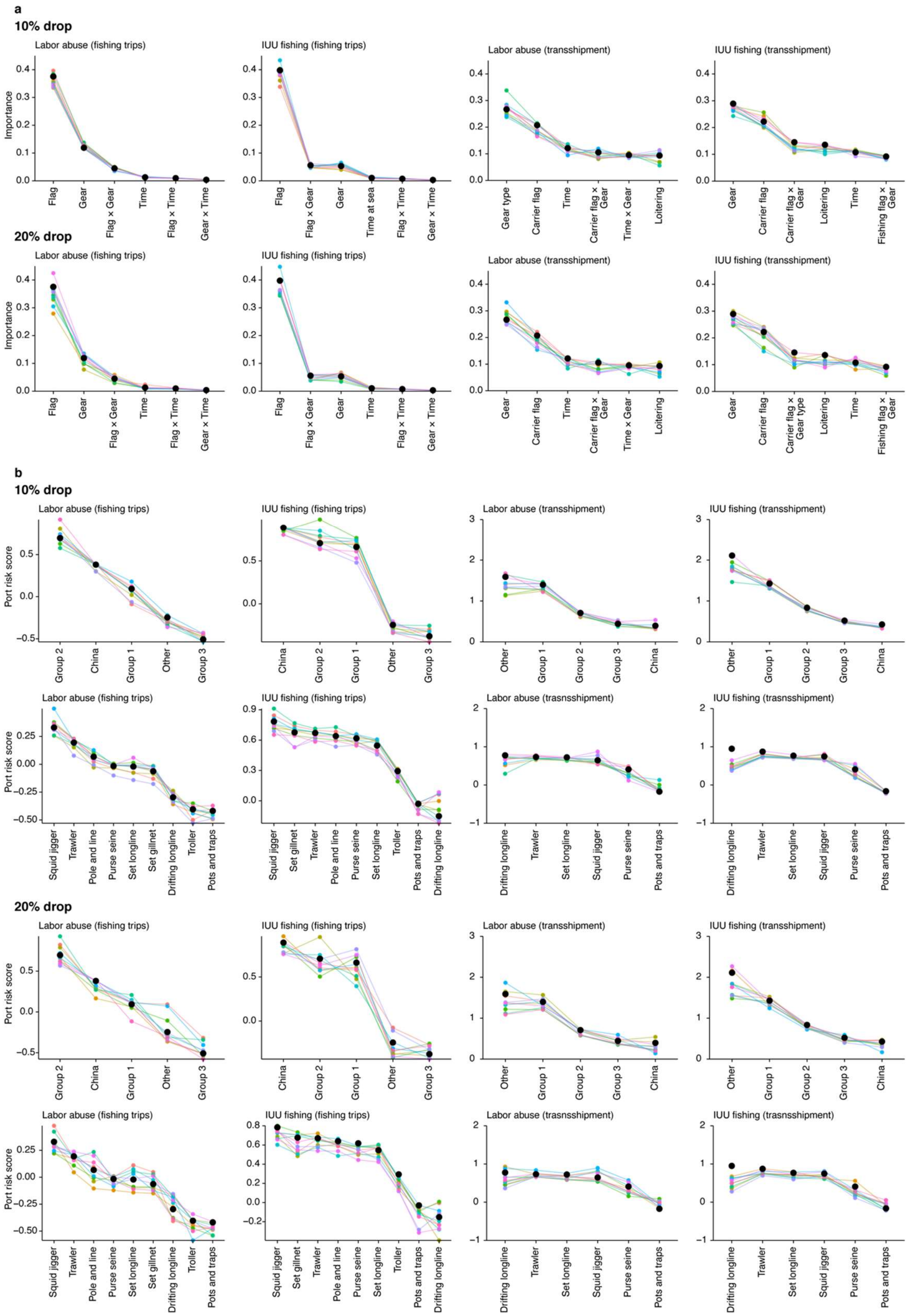

Supplementary Figure 4. Robustness of port risk scores. (**a**) Variable importance on port risk score for labor abuse and IUU fishing for fishing trips and transshipment after subsampling experts' responses on port risk assessment. (**b**) Effects of variables on port risk score for labor abuse and IUU fishing for fishing trips and transshipment after subsampling experts' responses on port risk assessment. Colors correspond to the results from the same subsampling set (10% or 20% random drop of the data). Black points indicate the original values from the full dataset.

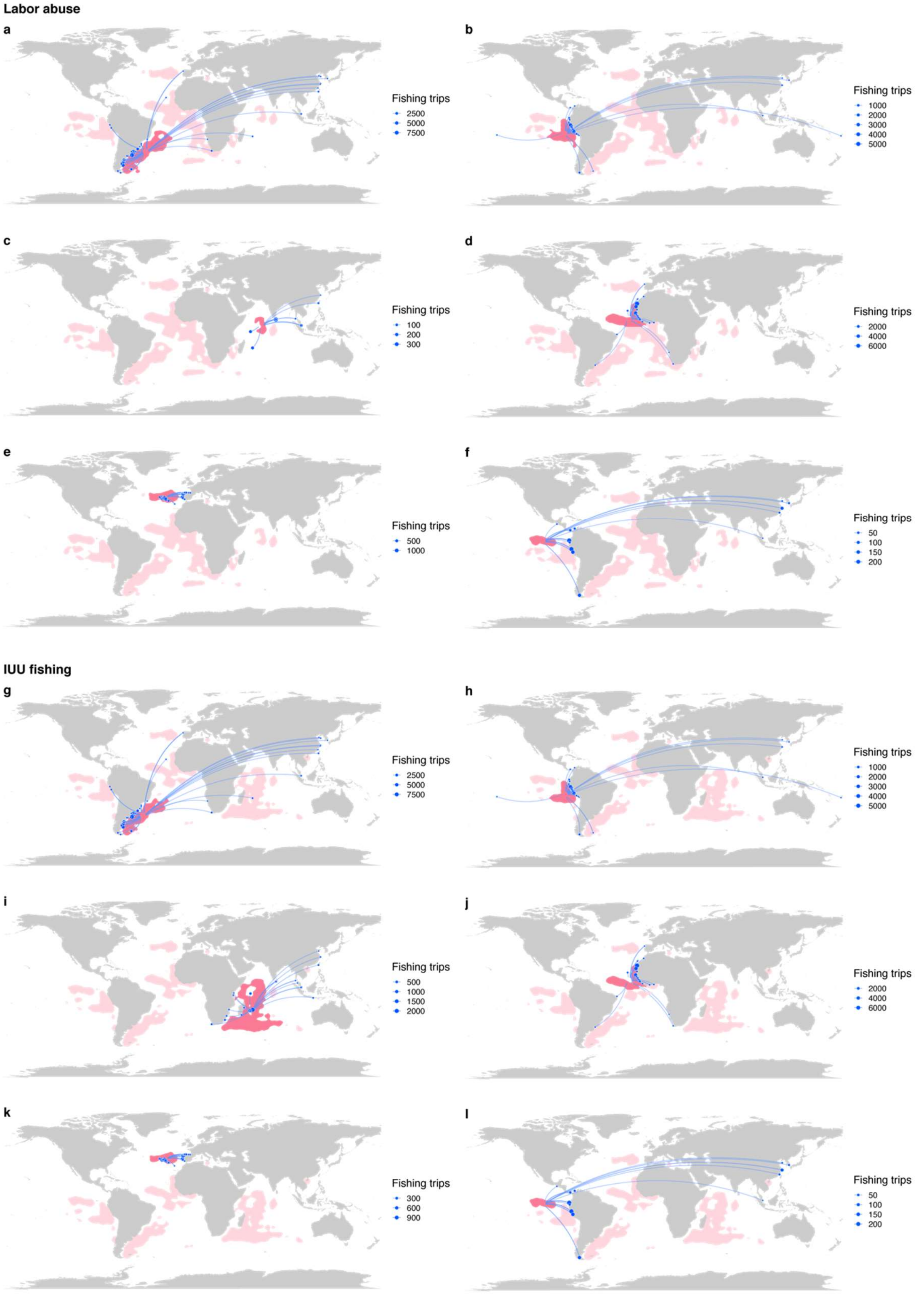

Supplementary Figure 5. Destination ports for high-risk fishing regions for labor abuse (a–f) and IUU fishing (g–l) (**a**) Southwest Atlantic, (**b**) Humboldt Current, (**c**) around the Maldives, (**d**) West Africa, (**e**) around the Azores, (**f**) around Galapagos Islands; (**g**) Southwest Atlantic (**h**) Humboldt Current, (**i**) Western Indian Ocean, (**j**) West Africa, (**k**) around the Azores, (**l**) around Galapagos Islands. Ports with at least 10 trips are shown.

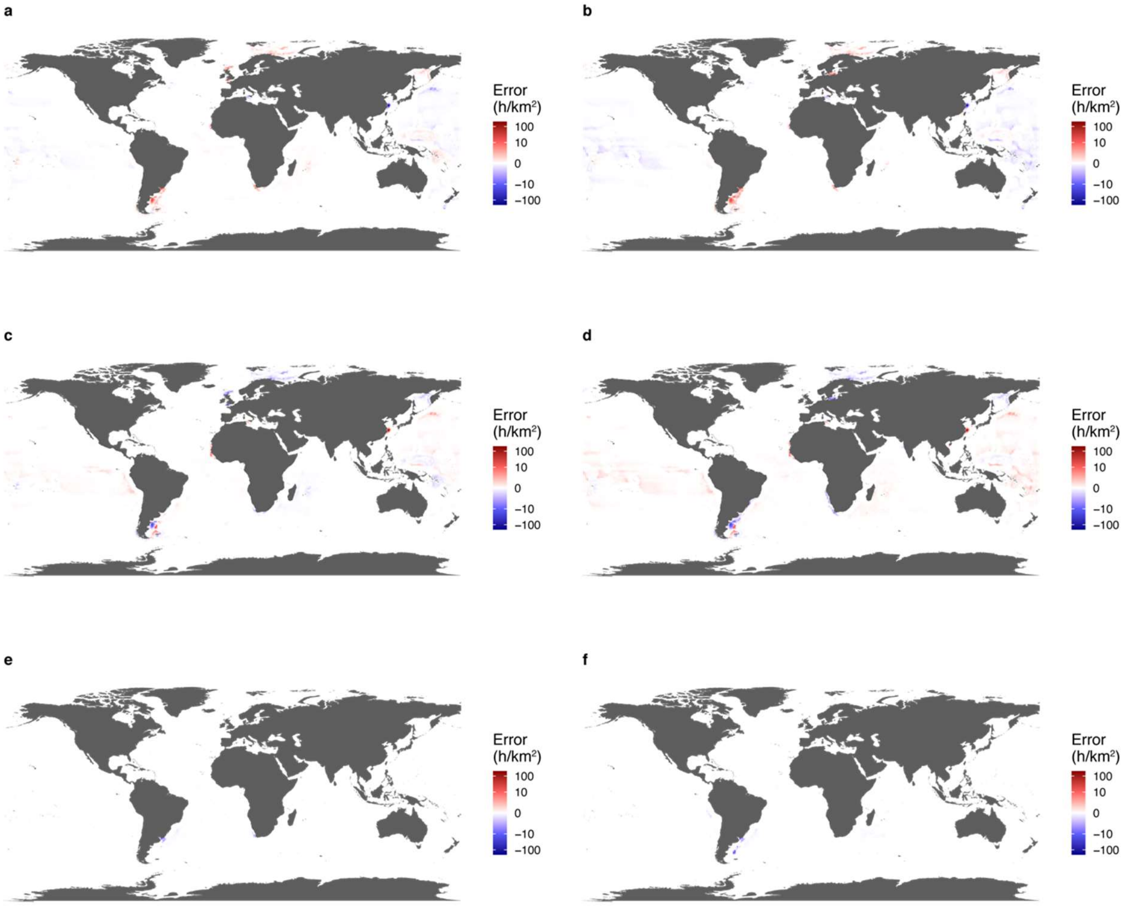

Supplementary Figure 6. Spatial differences between at-sea risk using the observed port risk score and the corresponding model prediction. The panels correspond to Supplementary Figure 1. The values represent cumulative fishing hours by area (hours/km^2) based on the predicted port risk score, minus the corresponding fishing hours based on the observed port risk score for low-risk ports for (**a**) labor abuse and (**b**) IUU fishing, medium-risk ports for (**c**) labor abuse and (**d**) IUU fishing, and high-risk ports for (**e**) labor abuse and (**f**) IUU fishing. Model errors on the observed port risk were mainly reflected in low- and medium-risk areas, with an overestimation (red range) of low-risk fishing off Uruguay, the Baltic Sea, and the Barents Sea and underestimation (blue range) of low-risk fishing in the Pacific region.

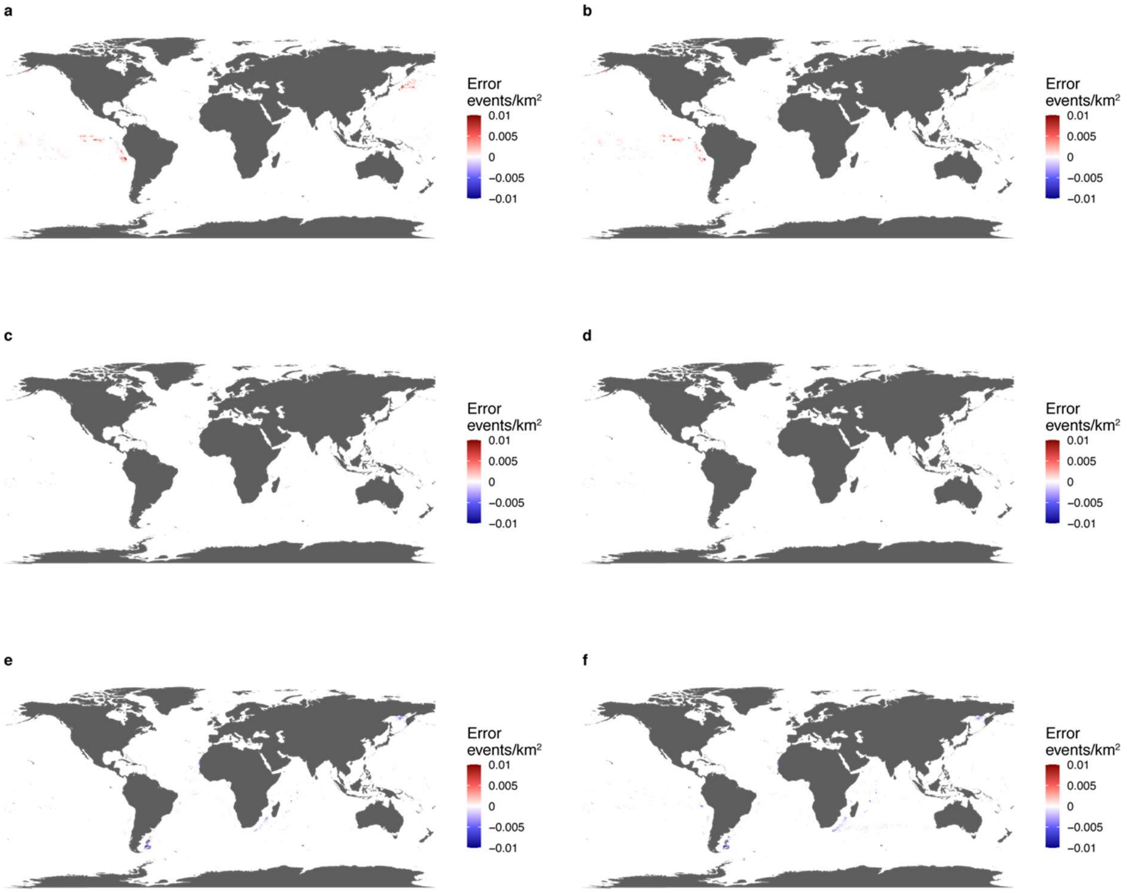

Supplementary Figure 7. Spatial difference between transshipment risk using the observed port risk score and the corresponding model prediction. The panels correspond to Supplementary Figure 3. The values represent density by area (no. of events/km^2) of encounter events based on the predicted port risk score, minus the corresponding density based on the observed port risk score for carrier vessels arriving at low-risk ports for (**a**) labor abuse and (**b**) IUU fishing, medium-risk ports for (**c**) labor abuse and (**d**) IUU fishing, and high-risk ports for (**e**) labor abuse and (**f**) IUU fishing. There is little visible spatial heterogeneity in error. Model errors on the observed port risk were mainly focused in low- and high-risk areas, with an overestimation (red range) of low-risk transshipments off Ecuador and an underestimation (blue range) of high-risk transshipments off Uruguay and South Africa.

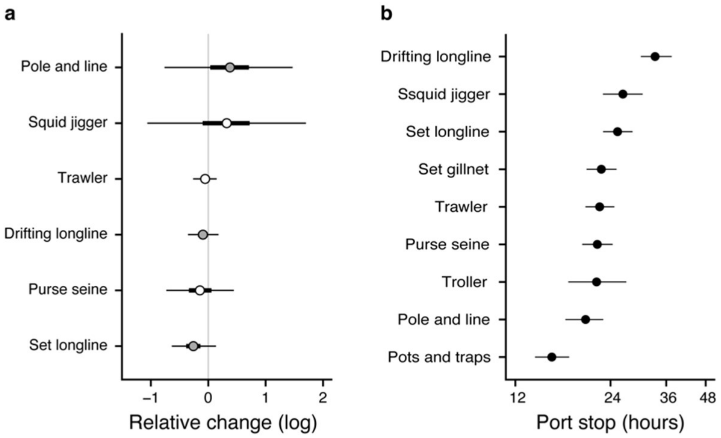

Supplementary Figure 8. Change in the number of visits by foreign fishing vessels to countries party to PSMA one year post entry into force relative to pre-PSMA period (a) and port stop duration of foreign fishing vessels by fishing gear types (**b**). In (**a**), points are medians of Bayesian posteriors, and thin and thick horizontal lines are 95% and 50% credible intervals (CIs), respectively. Grey points indicate that 50% CIs do not overlap with zero, and white points indicate that 50% CIs do overlap with zero. Pots and traps, trollers and set gillnets were removed from the analysis due to lack of convergence (< 20 observations). In (**b**), points and horizontal lines represent the best estimates and 95% confidence intervals, respectively.

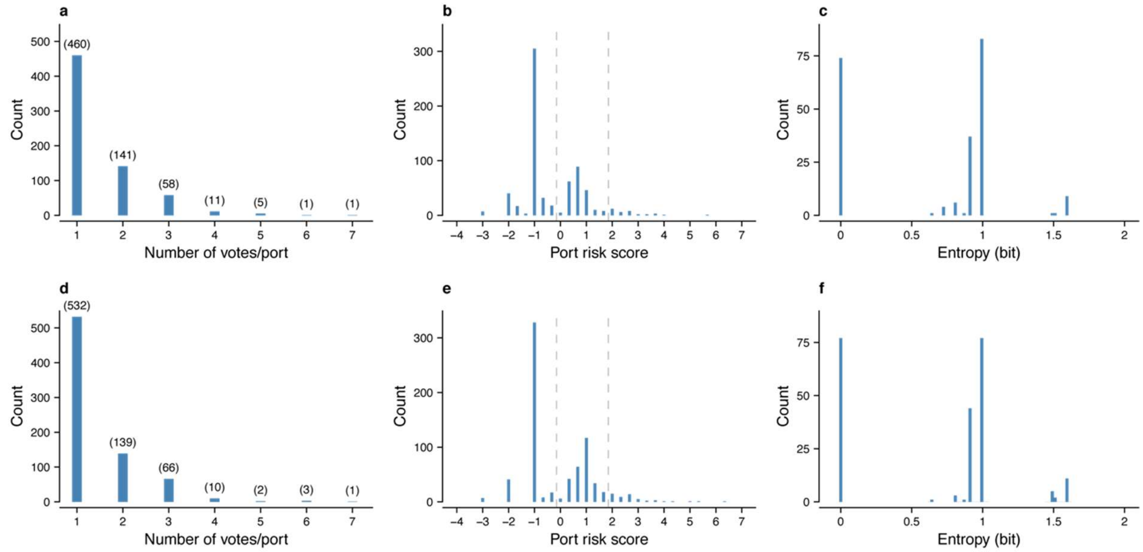

Supplementary Figure 9. Frequency of votes per port. Number of votes per port, port risk score, and entropy of votes ($n \geq 2$) for risk of labor abuse (**a–c**), and of IUU fishing (**d–f**). (**a, b**) Values in parentheses indicate frequency. (**b, e**) Port risk score was calculated as the sum of weighted votes for each port, where each vote was weighted by -1 for not associated, 1/3, 2/3 and 1 for low, medium and high degrees of certainty, respectively. Dashed vertical lines indicate clustering of port risk score into 3 bins (low: risk score < 0, medium: 0 ≤ risk score < 2, high: risk score ≥ 2) using a univariate k-means method based on the frequency distribution of combined port risk scores. (**c, f**) When a port received more than one vote, entropy was computed as $H = -\sum_{i=1}^{4} p_i \log_2 p_i$, where p_i represents a proportion of level i (low, medium, high, no association) over all votes. Entropy ranges from 0 to 2, with lower values indicating agreement among votes.

Supplementary Tables

Supplementary Table 1. Summary of the total numbers of respondents and ports per port assessment category for labor abuse and IUU fishing. The total number of ports assessed for labor abuse and IUU fishing includes ports that received multiple assessments with different degrees of certainty from experts so the total number of ports assessed are not equal to the sum of the ports under each category. Not all respondents who took the survey assessed ports for their level of risk, or for both labor abuse and IUU fishing.

	**Observations**
**Respondents**	**76**
**Ports assessed for labor abuse**	**677**
Not associated	432
Low degree of certainty	175
Medium degree of certainty	163
High degree of certainty	61
**Ports assessed for IUU fishing**	**753**
Not associated	432
Low degree of certainty	118
Medium degree of certainty	151
High degree of certainty	214

Supplementary Table 2. Flag categories used in the analysis, adapted from Ford and Wilcox[1]. China was excluded from the original flag group 3 in the analysis and considered separately given its dominance in fishing effort and number of vessels. Countries not included in either category were clustered under a separate class as 'other'. Flag group 1 corresponds to Cluster 2, Flag group 2 to Cluster 3, and Flag group 3 to Cluster 1 in the original analysis by Ford and Wilcox[1]. States in parentheses are listed in Ford and Wilcox[1], but lack associated AIS data in the 2012–2019 GFW dataset used for our analyses. We did not include Mongolia and Sao Tome and Príncipe because they were not in the subset of the Global Fishing Watch data we used for modeling. *Denotes a flag that is designated a Flag of Convenience according to the International Transport Workers' Federation[2].

Flag category	Ford and Wilcox description	State
**Flag group 1**	High ownership by countries other than the flag state; low fidelity to flag state EEZ, higher control of corruption	Antigua and Barbuda*, Barbados*, Cayman Islands*, Liberia*, Saint Vincent and the Grenadines*, Vanuatu*
**Flag group 2**	Low control of corruption; low fidelity to flag state EEZ; intermediate levels ownership by countries other than the flag state	Bahamas*, Bahrain, Belize*, Bolivia*, (Brunei Darussalam), Cambodia*, Cyprus*, Equatorial Guinea*, (Gabon), Georgia*, Honduras*, Kiribati, Madagascar, Malta*, Marshall Islands*, Panama*, Portugal, Saint Kitts and Nevis*, Samoa, Sierra Leone*, Sri Lanka*, (Tonga*), United Republic of Tanzania*
**Flag group 3**	Low ownership by countries other than the flag state; highest fidelity to flag state EEZ; intermediate control of corruption	Albania, Algeria, Angola, (Anguilla), Argentina, Australia, Azerbaijan, (Bangladesh), Belgium, Bermuda*, Brazil, Bulgaria, Cameroon*, Canada, Cape Verde, Chile, Colombia, (Congo), Costa Rica, Croatia, Cuba, Democratic People's Republic of Korea*, Denmark, Djibouti, Ecuador, Egypt, (Eritrea), Estonia, (Ethiopia), Fiji, Finland, France, Gambia, Germany, Ghana, Greece, Greenland, (Grenada), Guatemala, Guyana, Hong Kong, Iceland, India, Indonesia, Iran, (Iraq), Ireland, Israel, Italy, Japan, Jordan, Kazakhstan, Kenya, (Kuwait), (Lao People's Democratic Republic), Latvia, (Lebanon*), Libya, Lithuania, (Luxembourg), Malaysia, Maldives, Mauritania, Mauritius*, Mexico, Montenegro, Morocco, Mozambique, (Myanmar*), Namibia, Netherlands, New Zealand, Nigeria, Norway, Oman, Pakistan, Papua New Guinea, (Paraguay), Peru, Philippines, Poland, Qatar, Republic of Korea, Russian Federation, Saudi Arabia, Senegal, Seychelles, Singapore, Slovenia, South Africa, Spain, Sudan, (Suriname), Sweden, Switzerland, (Syrian Arab Republic), Taiwan, Thailand, (Trinidad and Tobago), Tunisia, Turkey, (Turkmenistan), Ukraine, (United Arab Emirates), United Kingdom, United States, Uruguay, Venezuela, Vietnam, (Yemen)
**China**	n/a	China
**Other**	n/a	Afghanistan, Aruba, Austria, Bhutan, Comoros*, Cook Islands*, Côte d'Ivoire, Curaçao*,

	Dominica, El Salvador, Falkland Islands, Faroe Islands*, French Guiana, Federated States of Micronesia, French Polynesia, French Southern Territories, Gibraltar*, Guinea, Guinea-Bissau, Hungary, Jamaica*, Lesotho, Moldova*, Nauru, New Caledonia, Nicaragua, Niue, Palau*, Palestine, Réunion, Romania, Saint Pierre and Miquelon, Serbia, Solomon Islands, Somalia, Togo*, Turks and Caicos Islands, Tuvalu, Wallis and Futuna

Supplementary Table 3. Summary of model input for fishing trips and risk of labor abuse, by gear type and for each flag category, at arrival ports. Values indicate the number of trips, total number of fishing hours and the number of unique ports in each flag group category. Values in parentheses represent the Shannon equitability index, calculated as $E = -\sum_{i=1}^{N} p_i \ln p_i / \ln N$, where N is the total number of unique ports and p_i is the proportion of the occurrence of port i within a flag × gear category. The score ranges from 0 to 1, with larger values indicating that frequencies of unique ports are more evenly distributed within a category. Flag groups are in Supplementary Table 2.

Gear type	Flag group 1	Flag group 2	Flag group 3	China	Other
Drifting longlines	550 trips<br>779,489 hours<br>12 ports<br>(*E* = 0.58)	577 trips<br>178,086 hours<br>24 ports<br>(*E* = 0.61)	32,578 trips<br>11,270,168<br>152 ports<br>(*E* = 0.57)	4,801 trips<br>4,163,521<br>40 ports<br>(*E* = 0.56)	624 trips<br>229,521 hours<br>22 ports<br>(*E* = 0.57)
Pole and line	7 trips<br>936 hours<br>3 ports<br>(*E* = 0.87)	NA	11,128 trips<br>192,992 hours<br>142 ports<br>(*E* = 0.6)	3,181 trips<br>52,707 hours<br>13 ports<br>(*E* = 0.64)	NA
Pots and traps	NA	1 trip<br>335 hours<br>1 port<br>(*E* = NA)	15,264 trips<br>329,662 hours<br>82 ports<br>(*E* = 0.70)	10,695 trips<br>36,379 hours<br>12 ports<br>(*E* = 0.47)	1 trip<br>< 1 hour<br>1 port<br>(E = NA)
Purse seines	47 trips<br>2,924 hours<br>9 ports<br>(*E* = 0.74)	1,032 trips<br>51,702 hours<br>37 ports<br>(*E* = 0.73)	79,097 trips<br>907,754 hours<br>238 ports<br>(*E* = 0.76)	21,453 trips<br>132,652 hours<br>34 ports<br>(*E* = 0.57)	1,394 trips<br>69,387 hours<br>44 ports<br>(*E* = 0.74)
Set gillnets	NA	135 trips<br>1,442 hours<br>3 ports<br>(*E* = 0.38)	83,343 trips<br>587,664 hours<br>120 ports<br>(*E* = 0.52)	180,537 trips<br>1,179,638<br>17 ports<br>(*E* = 0.58)	413 trips<br>950 hours<br>4 ports<br>(*E* = 0.69)
Set longlines	32 trips<br>222 hours<br>3 ports<br>(*E* = 0.63)	133 trips<br>1,905 hours<br>23 ports<br>(*E* = 0.63)	84,306 trips<br>2,024,528<br>221 ports<br>(*E* = 0.53)	125,122 trips<br>1,066,917<br>37 ports<br>(*E* = 0.52)	1,266 trips<br>174,550 hours<br>18 ports<br>(*E* = 0.26)
Squid jiggers	175 trips<br>18,115 hours<br>7 ports<br>(*E* = 0.82)	41 trips<br>10,037 hours<br>12 ports<br>(*E* = 0.91)	4,199 trips<br>669,775 hours<br>40 ports<br>(*E* = 0.63)	3,960 trips<br>1,827,458<br>29 ports<br>(*E* = 0.61)	NA
Trawlers	116 trips<br>10,141 hours<br>6 ports<br>(*E* = 0.26)	2,124 trips<br>144,368 hours<br>29 ports<br>(*E* = 0.48)	230,854 trips<br>11,298,989<br>299 ports<br>(*E* = 0.71)	893,306 trips<br>15,399,480<br>57 ports<br>(*E* = 0.47)	5,068 trips<br>640,282 hours<br>35 ports<br>(*E* = 0.58)
Trollers	NA	NA	2,850 trips<br>52,168 hours<br>36 ports<br>(*E* = 0.72)	384 trips<br>4,188 hours<br>10 ports<br>(*E* = 0.74)	3 trips<br>< 1 hour<br>1 port<br>(*E* = NA)

Supplementary Table 4. Summary of model input for fishing trips and risk of IUU fishing, by gear type and for each flag category, at arrival ports. Values indicate the number of trips, total number of fishing hours, the number of ports, and evenness of the frequency of the unique ports in each flag group category. Values in parentheses represent the Shannon equitability index, calculated as $E = -\sum_{i=1}^{N} p_i \ln p_i \,/\, \ln N$, where N is the total number of unique ports and p_i is the proportion of the occurrence of port i within a flag × gear category. The score ranges from 0 to 1, with larger values indicating that frequencies of unique ports are more evenly distributed within a category. Flag groups are in Supplementary Table 2.

Gear type	Flag group 1	Flag group 2	Flag group 3	China	Other
Drifting longlines	552 trips<br>785,486 hours<br>13 ports<br>(E = 0.57)	651 trips<br>231,204 hours<br>29 ports<br>(E = 0.64)	32,952 trips<br>11,570,660<br>160 ports<br>(E = 0.57)	5,162 trips<br>4,391,529<br>56 ports<br>(E = 0.57)	628 trips<br>229,521 hours<br>24 ports<br>(E = 0.56)
Pole and line	15 trips<br>2,332 hours<br>3 ports<br>(E = 0.84)	NA	11,868 trips<br>237,899 hours<br>145 ports<br>(E = 0.62)	13,267 trips<br>106,149 hours<br>31 ports<br>(E = 0.64)	NA
Pots and traps	NA	1 trip<br>335 hours<br>1 port<br>(E = NA)	13,586 trips<br>241,077 hours<br>82 ports<br>(E = 0.68)	14,294 trips<br>67,068 hours<br>32 ports<br>(E = 0.57)	1 trip<br>< 1 hour<br>1 port<br>(E = NA)
Purse seines	48 trips<br>2,924 hours<br>10 ports<br>(E = 0.73)	944 trips<br>45,101 hours<br>39 ports<br>(E = 0.72)	83,694 trips<br>924,874 hours<br>257 ports<br>(E = 0.76)	36,487 trips<br>193,688 hours<br>58 ports<br>(E = 0.67)	1,245 trips<br>61,566 hours<br>42 ports<br>(E = 0.72)
Set gillnets	NA	176 trips<br>2,437 hours<br>5 ports<br>(E = 0.55)	83,874 trips<br>580,807 hours<br>135 ports<br>(E = 0.52)	346,068 trips<br>2,779,464<br>38 ports<br>(E = 0.7)	413 trips<br>950 hours<br>4 ports<br>(E = 0.69)
Set longlines	36 trips<br>222 hours<br>4 ports<br>(E = 0.70)	231 trips<br>10,887 hours<br>29 ports<br>(E = 0.65)	84,390 trips<br>2,001,373<br>239 ports<br>(E = 0.52)	271,379 trips<br>2,259,801<br>62 ports<br>(E = 0.67)	1,259 trips<br>174,132 hours<br>17 ports<br>(E = 0.25)
Squid jiggers	211 trips<br>18,348 hours<br>10 ports<br>(E = 0.82)	46 trips<br>10,047 hours<br>15 ports<br>(E = 0.91)	5,710 trips<br>860,719 hours<br>46 ports<br>(E = 0.64)	5,449 trips<br>1,997,480<br>45 ports<br>(E = 0.66)	NA
Trawlers	483 trips<br>49,234 hours<br>9 ports<br>(E = 0.34)	2,270 trips<br>148,333 hours<br>32 ports<br>(E = 0.52)	259,144 trips<br>12,721,018<br>334 ports<br>(E = 0.71)	1,701,857<br>26,928,459<br>81 ports<br>(E = 0.60)	5,120 trips<br>636,932 hours<br>34 ports<br>(E = 0.60)
Trollers	NA	NA	2,851 trips<br>52,168 hours<br>37 ports<br>(E = 0.71)	716 trips<br>6,754 hours<br>25 ports<br>(E = 0.77)	3 trips<br>< 1 hour<br>1 port<br>(E = NA)

Supplementary Table 5. Summary of model input for carrier vessel trips that were classified as having encounters and risk of labor abuse at arrival ports, for each flag group and with and without loitering. Values indicate the number of trips by carrier vessels, the occurrence of loitering, the number of ports and the evenness of the frequency of unique ports in each flag group category as measured by the Shannon equitability index (see caption in Supplementary Tables 3 and 4 for how the index was calculated).

Loitering	Flag group 1	Flag group 2	Flag group 3	China	Other
Yes	233 trips<br>31 ports<br>(*E* = 0.79)	509 trips<br>41 ports<br>(*E* = 0.76)	1,514 trips<br>29 ports<br>(*E* = 0.38)	67 trips<br>8 ports<br>(*E* = 0.63)	47 trips<br>13 ports<br>(*E* = 0.90)
No	24 trips<br>6 ports<br>(*E* = 0.78)	39 trips<br>12 ports<br>(*E* = 0.80)	789 trips<br>29 ports<br>(*E* = 0.53)	2 trips<br>1 port<br>(*E* = NA)	5 trips<br>2 ports<br>(*E* = 0.97)

Supplementary Table 6. Summary of the model input for carrier vessel trips that were classified as having encounters and risk of IUU fishing at arrival ports, for each flag group and with and without loitering. The values indicate the number of trips by carrier vessels, the occurrence of loitering, the number of ports and the evenness of the frequency of the unique ports in each flag group category as measured by the Shannon equitability index (see caption in Supplementary Tables 3 and 4 for how the index was calculated).

Loitering	Flag group 1	Flag group 2	Flag group 3	China	Other
Yes	257 trips<br>34 ports<br>(*E* = 0.80)	555 trips<br>45 ports<br>(*E* = 0.77)	1,572 trips<br>32 ports<br>(*E* = 0.40)	79 trips<br>11 ports<br>(*E* = 0.70)	45 trips<br>12 ports<br>(*E* = 0.90)
No	24 trips<br>6 ports<br>(*E* = 0.78)	41 trips<br>14 ports<br>(*E* = 0.81)	806 trips<br>32 ports<br>(*E* = 0.54)	2 trips<br>1 port<br>(*E* = NA)	5 trips<br>2 ports<br>(*E* = 0.97)

Supplementary Table 7. Proportion of flag states over all known flag states in high-risk regions for labor abuse based on total fishing hours (2012–2019) for regions identified in Supplementary Figure 5: (**a**) Southwest Atlantic, (**b**) Humboldt Current, (**c**) around Maldives, (**d**) West Africa, (**e**) around the Azores, and (**f**) around the Galapagos Islands.

a

Flag State	Proportion
Argentina	0.59
Uruguay	0.23
Taiwan	0.03
Brazil	0.03
Spain	0.03
South Korea	0.03
Other	0.05

b

Flag State	Proportion
Peru	0.87
Other	0.13

c

Flag State	Proportion
Taiwan	0.45
Sri Lanka	0.31
China	0.10
Seychelles	0.07
South Korea	0.02
Other	0.05

d

Flag State	Proportion
Morocco	0.37
Spain	0.14
China	0.09
Senegal	0.07
Russia	0.03
Mauritania	0.03
Norway	0.02
Belize	0.02
Comoros	0.02
Guinea-Bissau	0.02
Italy	0.01
Curaçao	0.01
France	0.01
Japan	0.01
Cameroon	0.01
Portugal	0.01
Netherlands	0.01
South Korea	0.01
Indonesia	0.01
Turkey	0.01
Lithuania	0.01
Georgia	0.01
St. Kitts & Nevis	0.01
Other	0.05

e

Flag State	Proportion
Portugal	0.74
Other	0.26

f

Flag State	Proportion
China	0.57
Colombia	0.13
Ecuador	0.09
Panama	0.04
Spain	0.04
Venezuela	0.03
Taiwan	0.03
Other	0.07

Supplementary Table 8. Proportion of known flag states in high-risk regions for IUU fishing based on total fishing hours (2012–2019) for regions identified in Supplementary Figure 5: (**a**) Southwest Atlantic, (**b**) Humboldt Current, (**c**) Western Indian Ocean, (**d**) West Africa, (**e**) around the Azores, and (**f**) around the Galapagos Islands.

a

Flag State	Proportion
Argentina	0.59
Uruguay	0.23
Taiwan	0.03
Brazil	0.03
Spain	0.03
South Korea	0.03
Other	0.05

b

Flag State	Proportion
Peru	0.87
Other	0.13

c

Flag State	Proportion
Taiwan	0.29
France	0.25
Mauritius	0.11
China	0.08
Sri Lanka	0.06
Madagascar	0.05
Japan	0.03
Seychelles	0.03
South Korea	0.03
Other	0.07

d

Flag State	Proportion
Morocco	0.37
Spain	0.14
China	0.09
Senegal	0.07
Russia	0.03
Mauritania	0.03
Norway	0.02
Belize	0.02
Comoros	0.02
Guinea-Bissau	0.02
Italy	0.01
Curaçao	0.01
France	0.01
Cameroon	0.01
Japan	0.01
Netherlands	0.01
South Korea	0.01
Portugal	0.01
Indonesia	0.01
Turkey	0.01
Lithuania	0.01
Georgia	0.01
Other	0.05

e

Flag State	Proportion
Portugal	0.71
Other	0.29

f

Flag State	Proportion
China	0.58
Colombia	0.13
Ecuador	0.09
Panama	0.04
Spain	0.04
Venezuela	0.03
Taiwan	0.03
Other	0.07

Supplementary Table 9. Top 20 destination ports in high-risk regions for labor abuse (with number of trips ≥ 10) for regions identified in Supplementary Figure 5: (**a**) Southwest Atlantic, (**b**) Humboldt Current, (**c**) around Maldives, (**d**) West Africa, (**e**) around the Azores, and (**f**) around the Galapagos Islands. Port names are from Global Fishing Watch.

a

Country	Port	Trips	%
Uruguay	Montevideo	9,453	23.6
Argentina	Mar Del Plata	7,593	19.0
Argentina	Puerto Madryn	5,581	13.9
Argentina	Puerto Deseado	3,727	9.3
Argentina	Camarones	2,154	5.4
Brazil	Rio Grande	1,874	4.7
Falkland Islands	Stanley	1,335	3.3
Falkland Islands	Berkeley Sound	1,096	2.7
Argentina	Comodoro Rivadavia	990	2.5
Argentina	San Antonio Este	875	2.2
Argentina	Puerto Rawson	788	2.0
Argentina	Caleta Paula	556	1.4
Uruguay	Recalada	462	1.2
Uruguay	Punta Del Este	420	1.0
Brazil	Itajai	331	0.8
South Africa	Cape Town	286	0.7
Argentina	Caleta Olivia	262	0.7
Mauritius	Port Louis	207	0.5
Chile	Punta Arenas	196	0.5
Uruguay	La Paloma	194	0.5

b

Country	Port	Trips	%
Peru	Callao	5,442	19.6
Peru	Pisco	4,082	14.7
Peru	Chimbote	3,720	13.4
Peru	Coishco	3,162	11.4
Peru	Chicama	2,661	9.6
Peru	Chancay	1,592	5.7
Peru	Supe	1,376	4.9
Peru	Vegueta	1,359	4.9
Peru	La Pampilla	1,022	3.7
Peru	Tambo De Mora	968	3.5
Peru	Huacho	471	1.7
Peru	Samanco	416	1.5
Chile	Punta Arenas	283	1.0
Ecuador	Manta	191	0.7

Peru	La Punta	167	0.6
Peru	Tierra Colorada	146	0.5
China	Zhoushan	112	0.4
Peru	Bayovar	55	0.2
Ecuador	Posorja	43	0.2
French Polynesia	Papeete	41	0.1

c

**Country**	**Port**	**Trips**	**%**
Sri Lanka	Colombo	397	36.7
Seychelles	Victoria	151	14.0
Mauritius	Port Louis	138	12.8
Singapore	Singapore	126	11.6
Sri Lanka	Negombo	83	7.7
Taiwan	Kaohsiung	35	3.2
Thailand	Phuket	29	2.7
Sri Lanka	Hendala	24	2.2
Thailand	Ko He	20	1.8
Indonesia	Nipah Anchorage	15	1.4
China	Zhoushan	12	1.1

d

**Country**	**Port**	**Trips**	**%**
Western Sahara	Dakhla	7,400	42.2
Mauritania	Nouadhibou	4,071	23.2
Senegal	Dakar	3,339	19.0
Guinea	Conakry	443	2.5
Guinea-Bissau	Bissau	438	2.5
Cape Verde	Porto Grande	412	2.3
Sierra Leone	Freetown	256	1.5
Spain	Las Palmas	185	1.1
Côte d’Ivoire	Port Bouet	142	0.8
Côte d’Ivoire	Abidjan	106	0.6
Mauritania	Nouakchott	90	0.5
Brazil	Natal	78	0.4
Morocco	Agadir	57	0.3
Ghana	Tema	56	0.3
Senegal	Ziguinchor	51	0.3
Spain	Argineguin	45	0.3
Liberia	Monrovia	44	0.3
Spain	Cangas	40	0.2
South Africa	Cape Town	40	0.2

Gambia	Banjul	32	0.2

e

Country	Port	Trips	%
Portugal	Horta	1,410	15.5
Portugal	Ponta Delgada	1,317	14.4
Spain	Vigo	1,167	12.8
Spain	Cangas	1,012	11.1
Portugal	Praia De Vitoria	963	10.6
Portugal	Peniche	573	6.3
Portugal	Vila Do Porto	406	4.5
Portugal	Vila Franca Do Campo	267	2.9
Portugal	Madalena	238	2.6
Spain	Aviles	226	2.5
Spain	La Coruna	146	1.6
Spain	San Ciprian	145	1.6
Spain	Burela	119	1.3
Portugal	Sesimbra	119	1.3
Portugal	Praia	84	0.9
Portugal	Leixoes	83	0.9
Spain	Gijon	73	0.8
Spain	Bermeo	67	0.7
Portugal	Velas	61	0.7
Spain	Nerga	60	0.7

f

Country	Port	Trips	%
Ecuador	Manta	237	20.6
Peru	Chimbote	221	19.2
Peru	Callao	145	12.6
Chile	Punta Arenas	120	10.4
China	Zhoushan	99	8.6
Panama	Vacamonte	47	4.1
Ecuador	Posorja	35	3.0
Colombia	Cartagena	33	2.9
South Korea	Busan	28	2.4
China	Fuzhou	25	2.2
China	Weihai	16	1.4
Mexico	Puerto Madero	12	1.0
Singapore	Singapore	10	0.9

Supplementary Table 10. Top 20 destination ports in high-risk regions for IUU fishing (with number of trips ≥ 10) for regions identified in Supplementary Figure 5: (**a**) Southwest Atlantic, (**b**) Humboldt Current, (**c**) Western Indian Ocean, (**d**) West Africa, (**e**) around the Azores, and (**f**) around the Galapagos Islands. Port names are from Global Fishing Watch.

a

Country	Port	Trips	%
Uruguay	Montevideo	9,453	23.7
Argentina	Mar Del Plata	7,571	19.0
Argentina	Puerto Madryn	5,575	14.0
Argentina	Puerto Deseado	3,709	9.3
Argentina	Camarones	2,154	5.4
Brazil	Rio Grande	1,851	4.6
Falkland Islands	Stanley	1,319	3.3
Falkland Islands	Berkeley Sound	1,096	2.7
Argentina	Comodoro Rivadavia	988	2.5
Argentina	San Antonio Este	874	2.2
Argentina	Puerto Rawson	788	2.0
Argentina	Caleta Paula	555	1.4
Uruguay	Recalada	462	1.2
Uruguay	Punta Del Este	420	1.1
Brazil	Itajai	315	0.8
South Africa	Cape Town	283	0.7
Argentina	Caleta Olivia	262	0.7
Mauritius	Port Louis	207	0.5
Uruguay	La Paloma	194	0.5
Chile	Punta Arenas	193	0.5

b

Country	Port	Trips	%
Peru	Callao	5,428	19.7
Peru	Pisco	4,080	14.8
Peru	Chimbote	3,717	13.5
Peru	Coishco	3,159	11.5
Peru	Chicama	2,632	9.6
Peru	Chancay	1,592	5.8
Peru	Supe	1,376	5.0
Peru	Vegueta	1,359	4.9
Peru	La Pampilla	1,021	3.7
Peru	Tambo De Mora	966	3.5
Peru	Huacho	471	1.7
Peru	Samanco	416	1.5
Chile	Punta Arenas	282	1.0
Ecuador	Manta	181	0.7

Peru	Tierra Colorada	137	0.5
China	Zhoushan	109	0.4
Peru	La Punta	71	0.3
Peru	Bayovar	54	0.2
Ecuador	Posorja	43	0.2
Peru	Huarmey	39	0.1

c

Country	Port	Trips	%
Mauritius	Port Louis	2,038	37.3
Réunion	Port Ouest	1,210	22.1
Sri Lanka	Colombo	409	7.5
Seychelles	Victoria	341	6.2
Madagascar	Toamasina	254	4.6
Singapore	Singapore	221	4.0
South Africa	Durban	113	2.1
South Africa	Cape Town	86	1.6
Taiwan	Kaohsiung	82	1.5
Sri Lanka	Negombo	80	1.5
Réunion	Reunion	79	1.4
Indonesia	Tanjung Benoa	59	1.1
Mozambique	Maputo	42	0.8
Madagascar	Tamatave	35	0.6
Thailand	Phuket	30	0.5
China	Zhoushan	28	0.5
Mozambique	Beira	28	0.5
Sri Lanka	Hendala	26	0.5
Madagascar	Portl Ehoala	25	0.5
Indonesia	Nipah Anchorage	20	0.4
Thailand	Ko He	20	0.4

d

Country	Port	Trips	%
Western Sahara	Dakhla	7,400	42.5
Mauritania	Nouadhibou	4,070	23.4
Senegal	Dakar	3,331	19.1
Guinea	Conakry	443	2.5
Guinea-Bissau	Bissau	438	2.5
Cape Verde	Porto Grande	379	2.2
Sierra Leone	Freetown	255	1.5
Spain	Las Palmas	182	1.0
Côte d’Ivoire	Port Bouet	121	0.7

Côte d'Ivoire	Abidjan	94	0.5
Mauritania	Nouakchott	90	0.5
Morocco	Agadir	57	0.3
Brazil	Natal	54	0.3
Senegal	Ziguinchor	51	0.3
Ghana	Tema	50	0.3
Liberia	Monrovia	44	0.3
Spain	Argineguin	41	0.2
Spain	Cangas	40	0.2
South Africa	Cape Town	39	0.2
Gambia	Banjul	32	0.2

e

**Country**	**Port**	**Trips**	**%**
Portugal	Horta	1,172	15.8
Spain	Vigo	1,092	14.8
Spain	Cangas	961	13.0
Portugal	Praia De Vitoria	905	12.2
Portugal	Ponta Delgada	708	9.6
Portugal	Peniche	496	6.7
Spain	Aviles	225	3.0
Portugal	Madalena	214	2.9
Portugal	Vila Do Porto	175	2.4
Spain	San Ciprian	145	2.0
Spain	La Coruna	143	1.9
Spain	Burela	112	1.5
Portugal	Sesimbra	108	1.5
Portugal	Praia	83	1.1
Portugal	Leixoes	79	1.1
Spain	Gijon	68	0.9
Spain	Bermeo	66	0.9
Portugal	Vila Franca Do Campo	66	0.9
Portugal	Velas	61	0.8
Spain	Nerga	54	0.7

f

**Country**	**Port**	**Trips**	**%**
Ecuador	Manta	229	20.4
Peru	Chimbote	221	19.7
Peru	Callao	141	12.6
Chile	Punta Arenas	120	10.7
China	Zhoushan	99	8.8

Panama	Vacamonte	41	3.7
Ecuador	Posorja	34	3.0
Colombia	Cartagena	33	2.9
South Korea	Busan	26	2.3
China	Fuzhou	25	2.2
China	Weihai	16	1.4
Mexico	Puerto Madero	11	1.0
Singapore	Singapore	10	0.9

Supplementary Table 11. Proportion of gear types in high-risk regions for labor abuse based on total fishing hours (2012–2019) for regions identified in Supplementary Figure 5: (**a**) Southwest Atlantic, (**b**) Humboldt Current, (**c**) around Maldives, (**d**) West Africa, (**e**) around the Azores, and (**f**) around the Galapagos Islands.

a

Gear	Proportion
Trawlers	0.83
Squid jiggers	0.12
Other	0.05

b

Gear	Proportion
Purse seines	0.94
Other	0.06

c

Gear	Proportion
Drifting longlines	0.93
Other	0.07

d

Gear	Proportion
Trawlers	0.72
Purse seines	0.18
Other	0.10

e

Gear	Proportion
Drifting longlines	0.51
Pole and line	0.31
Set longlines	0.10
Other	0.08

f

Gear	Proportion
Squid jiggers	0.54
Purse seines	0.32
Other	0.14

Supplementary Table 12. Proportion of gear types in high-risk regions for IUU fishing based on total fishing hours (2012–2019) for regions identified in Supplementary Figure 5: (**a**) Southwest Atlantic, (**b**) Humboldt Current, (**c**) Western Indian Ocean, (**d**) West Africa, (**e**) around the Azores, and (**f**) around the Galapagos Islands

a

Gear	Proportion
Trawlers	0.83
Squid jiggers	0.12
Other	0.05

b

Gear	Proportion
Purse seines	0.95
Other	0.05

c

Gear	Proportion
Drifting longlines	0.87
Set longlines	0.05
Other	0.08

e

Gear	Proportion
Trawlers	0.72
Purse seines	0.18
Other	0.10

f

Gear	Proportion
Drifting longlines	0.58
Pole and line	0.23
Set longlines	0.11
Other	0.09

g

Gear	Proportion
Squid jiggers	0.55
Purse seines	0.32
Other	0.13

Supplementary Note 1

Robustness of port risk assessment

Overall, subsampling port risk scores from the port risk assessment did not substantially affect the results of variable importance and the effects on port risk score (Supplementary Figure 4). For fishing trips, different subsamples resulted in similar patterns of variable importance on the risk of labor abuse (ICC = 0.977, $F_{5, 53.9} = 445$ for 10% drop, ICC = 0.948, $F_{5, 53.8} = 188$ for 20% drop; $p < 0.001$ for both) and risk of IUU fishing (ICC = 0.986, $F_{5, 52.1} = 768$ for 10% drop, ICC = 0.986, $F_{5, 51.1} = 795$ for 20% drop; $p < 0.001$ for both), with greater importance of flag groups and gear types. The effects of variables on risk of labor abuse agreed among subsamples (ICC = 0.983, $F_{4, 40.9} = 701$ for 10% drop, ICC = 0.942, $F_{4, 39.6} = 201$ for 20% drop for flag groups; ICC = 0.966, $F_{8, 36.5} = 475$ for 10% drop, ICC = 0.924, $F_{8, 50.7} = 168$ for 20% drop for gear types; $p < 0.001$ for all). Similarly, flag groups and gear types showed similar effects on risk of IUU fishing (ICC = 0.985, $F_{4, 32.4} = 983$ for 10% drop, ICC = 0.967, $F_{4, 43.5} = 267$ for 20% drop for flag groups; ICC = 0.958, $F_{8, 29.6} = 431$ for 10% drop, ICC = 0.952, $F_{8, 34.0} = 339$ for 20% drop for gear types; $p < 0.001$ for all).

For transshipment, the random subsamples agreed on patterns of variable importance on risk of labor abuse (ICC = 0.971, $F_{14, 115} = 379$ for 10% drop, ICC = 0.970, $F_{14, 108} = 372$ for 20% drop; $p < 0.001$ for both) and risk of IUU fishing (ICC = 0.985, $F_{14, 76.8} = 870$ for 10% drop, ICC = 0.969, $F_{14, 104} = 370$ for 20% drop; $p < 0.001$ for both). Agreement was also attained in the effects of flag groups (ICC = 0.951, $F_{4, 42.1} = 223$ for 10% drop for labor abuse, ICC = 0.932, $F_{4, 43.2} = 203$ for 20% drop for labor abuse; ICC = 0.980, $F_{4, 41.1} = 577$ for 10% drop for IUU fishing, ICC = 0.953, $F_{4, 44.3} = 216$ for 20% drop for IUU fishing; $p < 0.001$ for all) and gear types (ICC = 0.914, $F_{5, 54} = 107$ for 10% drops labor abuse, ICC = 0.864, $F_{5, 43.3} = 83.5$ for 20% drop labor abuse; ICC = 0.961, $F_{5, 36.9} = 355$ for 10% drop for IUU fishing, ICC = 0.929, $F_{5, 33.2} = 204$ for 20% drop for IUU fishing; $p < 0.001$ for all).

Supplementary References

1 Ford, J. H. & Wilcox, C. Shedding light on the dark side of maritime trade – A new approach for identifying countries as flags of convenience. *Marine Policy* **99**, 298-303, doi:https://doi.org/10.1016/j.marpol.2018.10.026 (2019).

2 International Transport Workers' Federation. *Flags of Convenience*, 2020).
