# Supplementary material for: Revealing global risks of labor abuse and illegal, unreported, and unregulated fishing
Source: Nat Commun. 2022 Apr 5;13:1612. doi: 10.1038/s41467-022-28916-2 (PMC8983709; doi:10.1038/s41467-022-28916-2)
Supplement: Supplementary file 2 — Reporting Summary [file 41467_2022_28916_MOESM2_ESM.pdf]

nature research

Corresponding author(s): Elizabeth R Selig

Last updated by author(s): Jan 22, 2022

Reporting Summary

Nature Research wishes to improve the reproducibility of the work that we publish. This form provides structure for consistency and transparency in reporting. For further information on Nature Research policies, see our Editorial Policies and the Editorial Policy Checklist.

Statistics

For all statistical analyses, confirm that the following items are present in the figure legend, table legend, main text, or Methods section.

n/a	Confirmed	
☐	☒	The exact sample size (n) for each experimental group/condition, given as a discrete number and unit of measurement
☒	☐	A statement on whether measurements were taken from distinct samples or whether the same sample was measured repeatedly
☐	☒	The statistical test(s) used AND whether they are one- or two-sided<br>*Only common tests should be described solely by name; describe more complex techniques in the Methods section.*
☐	☒	A description of all covariates tested
☒	☐	A description of any assumptions or corrections, such as tests of normality and adjustment for multiple comparisons
☐	☒	A full description of the statistical parameters including central tendency (e.g. means) or other basic estimates (e.g. regression coefficient) AND variation (e.g. standard deviation) or associated estimates of uncertainty (e.g. confidence intervals)
☐	☒	For null hypothesis testing, the test statistic (e.g. F, t, r) with confidence intervals, effect sizes, degrees of freedom and P value noted<br>*Give P values as exact values whenever suitable.*
☐	☒	For Bayesian analysis, information on the choice of priors and Markov chain Monte Carlo settings
☐	☒	For hierarchical and complex designs, identification of the appropriate level for tests and full reporting of outcomes
☐	☒	Estimates of effect sizes (e.g. Cohen's d, Pearson's r), indicating how they were calculated

Our web collection on statistics for biologists contains articles on many of the points above.

Software and code

Policy information about availability of computer code

Data collection	We used the survey instrument Qualtrics and a satellite dataset of the AIS positions and vessel characteristics of fishing and support vessels curated by Global Fishing Watch. Data were extracted from GFW databases on September 15, 2021, and reflect updates to that date.
Data analysis	We used XGBoost v.1.0.0, shap v.0.35.0, and PyMC3 v.3.11.2 under Python 3.7.1, and lme4 v.1.1-26 under R 4.0.4. Custom code used in the analyses are available at: https://doi.org/10.5281/zenodo.5775311.

For manuscripts utilizing custom algorithms or software that are central to the research but not yet described in published literature, software must be made available to editors and reviewers. We strongly encourage code deposition in a community repository (e.g. GitHub). See the Nature Research guidelines for submitting code & software for further information.

Data

Policy information about availability of data

All manuscripts must include a data availability statement. This statement should provide the following information, where applicable:

- Accession codes, unique identifiers, or web links for publicly available datasets
- A list of figures that have associated raw data
- A description of any restrictions on data availability

Due to the sensitive nature of the port data, the specificity of the geographic information, and the associated ethics considerations, raw port data cannot be made publicly available. Requests for access to the data may be negotiated through a data use agreement and will need to be consistent with the ethics protocol and conditions of informed consent. Please contact Elizabeth Selig (eselig@stanford.edu) for more details.
The AIS vessel location data that support the findings of this study are available from Global Fishing Watch https://globalfishingwatch.org/.
All other data that were used to produce the results, including ratification dates of PSMA and C188 as well as fishing vessel and carrier vessel hold capacity data are available at: https://doi.org/10.5281/zenodo.5775311.

Field-specific reporting

Please select the one below that is the best fit for your research. If you are not sure, read the appropriate sections before making your selection.

☐ Life sciences ☒ Behavioural & social sciences ☐ Ecological, evolutionary & environmental sciences

For a reference copy of the document with all sections, see nature.com/documents/nr-reporting-summary-flat.pdf

Behavioural & social sciences study design

All studies must disclose on these points even when the disclosure is negative.

Study description	The study is mixed-methods, combining an online expert survey with a curated dataset on the position and vessel characteristics of fishing and support vessels from satellite data to estimate risks of labor abuse and illegal, unreported and unregulated fishing.
Research sample	We developed a survey on port risks and the relationships between labor abuse and IUU fishing and translated it into French and Spanish for a wider geographic sample. We assembled a respondent list that included potential experts on fishing, IUU fishing, labor abuse and human rights, from academia, civil society, business and government, based on internet searches, conference proceedings, and academic literature. Because the study was global, we attempted to identify at least 10 respondents per country. Between different survey distributions, we identified additional respondents in poorly sampled regions. The survey was sent to approximately 840 respondents. Respondents were encouraged to forward the survey to colleagues or those with relevant knowledge, so we cannot know precisely how many people received the survey.
Sampling strategy	We sent the survey to respondents directly, but used a snowball sampling approach to allow respondents to forward the survey to colleagues. We tested robustness of the port risk scores (the primary data collected by the survey) by dropping 10% and 20% of the survey data (Supplementary Figure 9) to determine if scores changed substantially, which they did not. Each time we opened the survey to respondents, we extended the date, with IRB approval, if we were still getting more than 1-2 responses per day. No predetermined sample size was calculated because without prior data on labor abuse and IUU fishing risk at port, data gained from experts was additive, i.e. all information was relevant as long as robustness metrics determined that modeling was not affected by smaller sample sizes.
Data collection	We used the online survey program Qualtrics to collect the survey data. No personally identifiable information was collected, including IP addresses.
Timing	We circulated the survey at three different time points with different distribution lists: January 30 - April 5, 2019, June 15 – August 5, 2019, and September 5 - 25, 2019.
Data exclusions	No data were excluded, expect for quality control, as specified in the Methods of the manuscript.
Non-participation	Because of snowball sampling, i.e., the survey link could be anonymously forwarded, we cannot know how many people received the survey and declined participation. We estimate the response rate to be 10-15% of those contacted.
Randomization	No experimental groups were used.

Reporting for specific materials, systems and methods

We require information from authors about some types of materials, experimental systems and methods used in many studies. Here, indicate whether each material, system or method listed is relevant to your study. If you are not sure if a list item applies to your research, read the appropriate section before selecting a response.

Materials & experimental systems

n/a	Involved in the study	
☒	☐	Antibodies
☒	☐	Eukaryotic cell lines
☒	☐	Palaeontology and archaeology
☒	☐	Animals and other organisms
☐	☒	Human research participants
☒	☐	Clinical data
☒	☐	Dual use research of concern

Methods

n/a	Involved in the study	
☒	☐	ChIP-seq
☒	☐	Flow cytometry
☒	☐	MRI-based neuroimaging

Human research participants

Policy information about studies involving human research participants

Population characteristics	The survey was anonymous, so the data collected did not include personally identifiable information. We did collect data on the sector that the expert works in.
Recruitment	We developed a respondent list that included potential experts from academia, civil society, business and government on fishing, IUU fishing, labor abuse and human rights. Because the survey was anonymous, we cannot assess to what extent the respondents' propensity for participation correlated with their knowledge of the researchers, specific responses, questions, and topics. For example, people who were familiar with both topics may have been more likely to complete the survey than people who were familiar with just one of the topics.
Ethics oversight	The survey study was approved by the Stanford Institutional Review Board (IRB #49308). Within the survey instrument, all participants were provided with information about the study and advised that their participation was entirely voluntary and anonymous (i.e. that responses could not be tracked to an individual). They were also advised how they could contact Stanford IRB with any concerns. Informed consent was documented by tick-box to ensure anonymity, because signature would have provided identifiable information. Participants were also advised of withdrawal procedures, and that they could opt out of taking the survey at any time by closing their browser.

Note that full information on the approval of the study protocol must also be provided in the manuscript.
